# Blood-Brain Barrier and Delivery of Protein and Gene Therapeutics to Brain

**DOI:** 10.3389/fnagi.2019.00373

**Published:** 2020-01-10

**Authors:** William M. Pardridge

**Affiliations:** Department of Medicine, University of California, Los Angeles, Los Angeles, CA, United States

**Keywords:** blood-brain barrier, cerebrospinal fluid, Alzheimer’s disease, Trojan horse, endothelium

## Abstract

Alzheimer’s disease (AD) and treatment of the brain in aging require the development of new biologic drugs, such as recombinant proteins or gene therapies. Biologics are large molecule therapeutics that do not cross the blood-brain barrier (BBB). BBB drug delivery is the limiting factor in the future development of new therapeutics for the brain. The delivery of recombinant protein or gene medicines to the brain is a binary process: either the brain drug developer re-engineers the biologic with BBB drug delivery technology, or goes forward with brain drug development in the absence of a BBB delivery platform. The presence of BBB delivery technology allows for engineering the therapeutic to enable entry into the brain across the BBB from blood. Brain drug development may still take place in the absence of BBB delivery technology, but with a reliance on approaches that have rarely led to FDA approval, e.g., CSF injection, stem cells, small molecules, and others. CSF injection of drug is the most widely practiced approach to brain delivery that bypasses the BBB. However, drug injection into the CSF results in limited drug penetration to the brain parenchyma, owing to the rapid export of CSF from the brain to blood. A CSF injection of a drug is equivalent to a slow intravenous (IV) infusion of the pharmaceutical. Given the profound effect the existence of the BBB has on brain drug development, future drug or gene development for the brain will be accelerated by future advances in BBB delivery technology in parallel with new drug discovery.

## Introduction

Progress in the development of new pharmaceuticals for the treatment of the brain in aging, including Alzheimer’s disease (AD), has been slow because the products of biotechnology, recombinant proteins or gene therapies, are all large molecule pharmaceuticals that do not cross the blood-brain barrier (BBB). In 2019, there is not a single recombinant protein that is FDA approved for the treatment of AD, or any other brain disease, wherein the drug must cross the BBB to enter the brain. Bevacizumab (Avastin^®^) is FDA approved for brain cancer (Han et al., 2014), but this monoclonal antibody (MAb) does not cross the non-disrupted BBB (Liu et al., 2016), and works by sequestration of vascular endothelial growth factor (VEGF) within the blood volume of the tumor. Natalizumab (Tysabri^®^) is FDA approved for the treatment of multiple sclerosis (Cadavid et al., 2013), but this MAb does not cross the BBB and works by blocking the trafficking of lymphocytes across the brain endothelial wall (Engelhardt and Coisne, 2011). In 2019, an adeno-associated virus (AAV)-9-based gene therapy was approved for the treatment of infantile spinal muscular atrophy (SMA)-1 with a 1-time intravenous (IV) administration of the self-complementary (sc)-AAV9 encoding the survival motor neuron type 1 (SMN1) gene (Mendell et al., 2017). This is the first FDA approved biologic for a brain disease that crosses the BBB. The development of this new treatment for SMA did not arise from a rational drug delivery platform, but rather was a product of the serendipitous finding that the AAV9 serotype traverses the BBB (Foust et al., 2009). However, as discussed below, there are limitations to the delivery of therapeutic genes to the brain with the AAV platform.

The discovery that certain AAV serotypes, such as AAV9, undergo transport across the BBB is a model of past efforts in the discovery of BBB-penetrating brain pharmaceuticals. That is, transport across the BBB was discovered serendipitously, rather than being an outgrowth of a focused effort in BBB transport technology. L-DOPA was developed as a treatment for Parkinson’s disease (PD) after the incidental finding that this amino acid precursor of dopamine, the neurotransmitter deficient in PD, penetrated the BBB following systemic administration (Davidson et al., 1971). L-DOPA is a large neutral amino acid and the mechanism of L-DOPA transport across the BBB was subsequently shown to be carrier-mediated transport *via* the BBB large neutral amino acid transporter (Wade and Katzman, 1975), and L-DOPA transport is mediated *via* the large neutral amino acid transporter type 1 (LAT1; Kageyama et al., 2000). The LAT1 transporter is selectively expressed at the BBB (Boado et al., 1999). Similarly, gabapentin, a cyclic gamma-amino acid, was discovered to penetrate the brain (Vollmer et al., 1986), and this was later shown to be mediated by the LAT1 transporter at the BBB (Dickens et al., 2013).

The brain drug development of brain-penetrating small molecules, recombinant proteins, or gene medicines is generally not successful unless the pharmaceutical crosses the BBB. Brain drug development should be executed as a binary process with parallel advancement in both brain drug discovery and BBB drug delivery, but this has not been the practice of the pharmaceutical industry. What has taken place is a primary focus on brain drug discovery with a minimal, if any, parallel effort in BBB drug delivery. As a consequence, there are few present-day FDA approved biologics for the serious diseases of the brain in aging, such as AD or PD. This situation is not expected to change for at least a generation if current practices continue and brain drug development operates in a BBB-free zone. This review will outline present-day approaches to brain drug delivery and discuss a platform for future brain drug development that merges BBB drug delivery technology with brain drug and gene discovery.

## Biologic Treatments of Alzheimer’s Disease Illustrate the Need for BBB Drug Delivery

The dementia of AD is caused by the deposition of Abeta amyloid plaque in the brain (Cummings and Cotman, 1995; Näslund et al., 2000), which arises from the abnormal processing of the amyloid precursor protein (APP). The amyloid hypothesis emerged in 1984, following the isolation and N-terminal amino acid sequencing of the 43 amino acid Abeta amyloid peptide, which has a single carboxyl-terminal threonine residue (Glenner and Wong, 1984). In the intervening 35 years, there is not a single FDA approved therapeutic for AD that reverses the dementia of AD secondary to intervention in the formation of the Abeta plaque. Clinical trials of drugs, such as anti-amyloid antibodies (AAA), aimed at the reduction of amyloid plaque in the brain in AD have not led to FDA approval. This dismal record in AD drug development has led many to question the validity of the amyloid hypothesis of AD (Panza et al., 2019). However, the failure of these clinical trials should not be used to refute the validity of the amyloid hypothesis of AD, if the anti-amyloid drug never reached the intended target owing to lack of BBB transport. If a drug aimed at brain amyloid, or any other target in the brain, does not cross the BBB, then no success in the clinical trial can be expected, and the amyloid hypothesis of AD has not been tested by the clinical trial. Multiple AAAs have failed in AD clinical trials because in all cases, the drugs do not cross the BBB, and no BBB drug delivery technology was introduced in AAA drug development (Pardridge, 2019).

**Figure 1:**
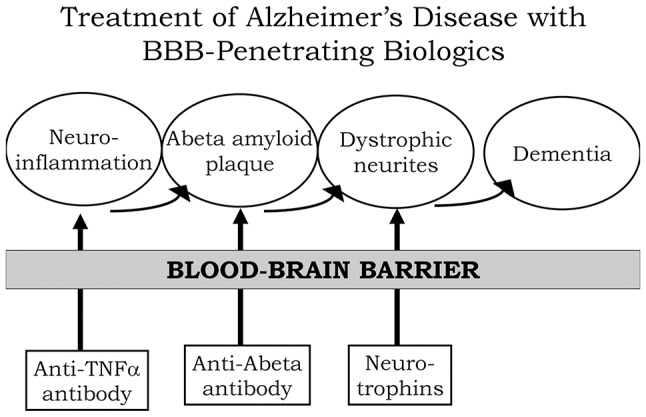
Model for combination therapy of Alzheimer’s disease (AD) with blood-brain barrier (BBB)-penetrating biologic drugs is based on blocking the pathway leading to dementia at multiple levels within the brain. Pro-inflammatory cytokines in the brain, such as tumor necrosis factor (TNF)-α, cause neuro-inflammation, and this inflammation may accelerate Abeta amyloid peptide plaque formation. Plaque formation causes neurite dystrophy, leading to dementia. BBB-penetrating biologic drugs are re-engineered as IgG Trojan horse fusion proteins. Biologic TNF inhibitors, such as TNFα decoy receptors or anti-TNFα therapeutic antibodies, are respectively re-engineered as a BBB penetrating IgG-decoy receptor fusion protein or as a bispecific antibody. Therapeutic antibodies that disaggregate the Abeta amyloid plaque are re-engineered as Trojan horse bispecific antibodies. Neurotrophins are re-engineered as IgG-neurotrophin fusion proteins (Figure 4).

A process by which amyloid plaque accumulation in the brain in AD leads to dementia is depicted in Figure 1. Dementia and cognitive decline of AD is not caused by amyloid plaque, *per se*, because amyloid plaque accumulation occurs before the onset of cognitive decline; the amyloid plaque leads to neurite dystrophy and synaptic loss, which then leads to dementia (Serrano-Pozo et al., 2011). If plaque reduction is not followed by repair of dystrophic neurites, then no improvement in dementia can be expected. AD is a chronic neuro-inflammatory condition mediated by proinflammatory cytokines, such as tumor necrosis factor (TNF)-α (McAlpine and Tansey, 2008). Therefore, a reversal of plaque formation may be followed by the further accumulation of plaque if the underlying neuroinflammation in the brain is not treated (Figure 1). Biologic drugs are currently available that can intervene in all three steps causing the dementia of AD (Figure 1). Neuro-inflammatory cytokines, such as TNFα, can be blocked by delivery to the brain of biologic TNF inhibitors (TNFI), such as anti-TNFα antibodies (Humira^®^, Remicade^®^), or TNFα decoy receptors (Enbrel^®^). However, these biologic TNFIs do not cross the BBB (Pardridge, 2010), and industry has made little attempt to re-engineer these agents for BBB delivery. The amyloid plaque of AD can be reduced by AAAs following direct intracerebral injection of the antibody (Solomon et al., 1997). In these early studies, the AAA was injected directly into the brain, because the AAA does not cross the BBB. However, the clinical trials for AAAs, e.g., bapineuzumab, solanezumab, gantenerumab, aducanumab, and others, administered the AAA by IV infusion on the assumption that the AAA crossed the BBB. When BBB transport of AAAs is measured, no BBB transport is observed in the absence of BBB delivery technology (Boado et al., 2007). The AAAs entered AD clinical trials with IV infusion, because industry made no attempt to develop BBB delivery technology to enable the re-engineering of these AAAs for BBB transport. With respect to repair of dystrophic neurites, neurotrophins, such as brain-derived neurotrophic factor (BDNF), ciliary neurotrophic factor (CNTF), fibroblast growth factor (FGF)-2, and others, can enhance neuronal repair in neurodegeneration (Kazim and Iqbal, 2016). However, the BDNF, CNTF, or FGF2 clinical trials for brain disease failed, because these agents do not cross the BBB (Pardridge, 2015a). What is needed for the treatment of the dementia of AD is a combination drug therapy that simultaneously aims to reduce neuroinflammation in the brain, disaggregate amyloid plaque, and repair dystrophic neurites. However, in the case of all three classes of biologic drugs for the brain, the neurotherapeutic needs to be re-engineered for BBB drug delivery (Figure 1). Such re-engineering platforms are available as discussed below in the context of molecular Trojan horses for BBB drug delivery of biologics. Prior to the discussion of transvascular drug delivery to the brain, it is necessary to review intra-thecal drug delivery to the brain, since this has been the default approach to brain drug delivery for decades.

## Brain Drug Delivery Into Cerebrospinal Fluid

### Bulk Flow of Cerebrospinal Fluid Within the Brain

The surface of the human brain is bathed with about 140 ml of cerebrospinal fluid (CSF). The CSF is secreted by the choroid plexus at each of the four ventricles of the brain (two lateral ventricles (LVs), a 3rd ventricle, and a 4th ventricle), and the entire volume of CSF is produced every 4–5 h or 4–5 times per day in the human brain (Pardridge, 2016). This CSF is rapidly exported to the blood *via* absorption into the superior sagittal sinus across the arachnoid villi. Owing to this rapid egress of CSF from brain to blood, intrathecal injection of the drug into CSF is similar to a slow IV infusion (Fishman and Christy, 1965). In contrast to the rapid rate of bulk flow of CSF out of the cranium, the diffusion of the drug into brain parenchyma from the CSF is limited, because diffusion decreases with the square of the diffusion distance. Consequently, drug is distributed only to the ependymal surface of brain following injection into CSF, and not into the brain parenchyma (Pardridge, 2016). The intra-thecal route of drug delivery to brain is suitable for treatment of diseases that affect the surface of the brain, such as carcinomatous meningitis, but is not able to deliver drug into the parenchyma of brain without exposing the surface of the brain to high, and generally toxicologic, drug concentrations (Yamada et al., 1991; Day-Lollini et al., 1997).

### Different Routes of Drug Injection Into CSF: Lumbar, Ventricular, or Cisternal

The simplest route of drug injection into CSF is a lumbar puncture. However, MRI studies in the primate show this route of delivery may treat the surface of the spinal cord, but very little drug reaches the cerebral hemispheres (Ohno et al., 2019). An alternative route of CSF injection is intra-cerebroventricular (ICV) delivery following the implantation of an Ommaya reservoir into one of the two LVs (Ommaya, 1963). Recently FDA approval was granted for the treatment of Batten disease type 2, a childhood lysosomal storage disorder caused by mutations in the gene encoding the lysosomal enzyme, tripeptidyl peptidase (TPP)-1, by ICV delivery using an Ommaya reservoir (Schulz et al., 2018). Recombinant human TPP1 (Brineura^®^) is infused into one LV every 2 weeks. Approval was granted because of improvement in peripheral motor function, although no effect of drug treatment on dementia, seizures, or blindness of Batten disease type 2 was recorded. The TPP1 enzyme is taken up by peripheral tissues *via* the mannose 6-phosphate receptor (M6PR), and delivery of TPP1 to peripheral tissues following injection into an Ommaya reservoir would be expected as the enzyme passes rapidly from CSF to peripheral blood, and then to the M6PR of peripheral tissues. A peripheral mechanism of action cannot be excluded, because there was no control arm in the clinical trial that administered the TPP1 enzyme by IV infusion (Schulz et al., 2018). Apart from rapid drug delivery to the peripheral bloodstream, other limitations of the Ommaya reservoir include the poor distribution of the drug to the contralateral side of the brain, owing to minimal retrograde flux of CSF from the 3rd ventricle to the opposite LV (discussed below). The third route of CSF injection that achieves the best distribution of drugs to both the bilateral forebrain and the spinal cord is a cisternal injection in the cerebro-medullary cistern (CMC; Ohno et al., 2019). However, this route of injection is technically difficult, is near vital structures of the brain, and has yet to enter into clinical practice. Still, a CMC injection would be expected to deliver the drug into brain parenchyma by reliance on drug diffusion from the CSF surface of the brain.

### Diffusion as the Primary Mechanism for Drug Penetration Into the Brain From CSF

**Figure 2:**
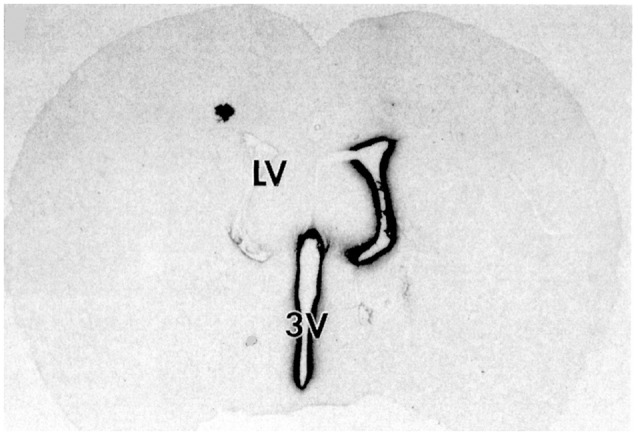
Whole-brain autoradiography study shows the limited penetration of biologic drugs into brain parenchyma following drug injection into the lateral ventricle (LV) in the rat. [^125^I]-brain derived neurotrophic factor (BDNF) was injected into the LV and the brain removed 20 h later for autoradiography. The BDNF moves from the LV to the third ventricle (3V), then to the fourth ventricle, over the surface of the brain, and is absorbed into the blood of the superior sagittal sinus. The drug only distributes into ~0.2 mm of brain parenchyma ipsilateral to the injection with no measurable distribution to the contralateral brain. Reprinted by permission from Yan et al. (1994).

Following drug injection into the CSF compartment, the drug may gain access to brain parenchyma *via* one of two mechanisms: (a) diffusion; or (b) bulk flow through perivascular spaces (Pardridge, 2016). If diffusion is the primary mechanism, then a logarithmic decline in drug concentration would be expected as drug diffuses into the brain from the CSF compartment; this is because diffusion decreases with the square of the diffusion distance. Conversely, if bulk flow through perivascular spaces was the predominant pathway, then a more uniform distribution of the drug in the brain would be observed with minimal decline in drug concentration within the parenchyma as compared to the drug concentration in CSF. The rate of bulk flow through perivascular space is low, 0.2 μl/min, and only about 5% the rate of CSF flow in the rat, 3.4 μl/min (Pardridge, 2016). The evidence accrued over the last 40 years of research on CSF drug delivery to brain indicates diffusion is the primary mechanism for drug movement into the brain from the CSF surface. Blasberg et al. (1975) injected small molecule drugs into one LV of the primate, and then measured the drug concentration at each mm of brain removed from the CSF surface. A steep logarithmic decline in drug concentration in brain parenchyma was observed. The concentration in the brain of thiotepa, a small molecule, was only 1% of the CSF concentration at just 1 mm of distance removed from the CSF surface. The poor distribution of drugs into brain parenchyma following injection into a LV is illustrated with brain autoradiography (Figure 2). The distribution of BDNF in rat brain was measured with autoradiography of brain obtained 20 h after the ICV injection of [^125^I]-BDNF in the LV (Yan et al., 1994). As shown in Figure 2, the BDNF has diffused only 0.2 mm into the parenchyma ipsilateral to the LV injection, with no measurable BDNF in the contralateral brain. This is because after drug is injected into an LV, the drug moves by CSF bulk flow to the third ventricle (3V), with minimal reflux up into the contralateral LV, then moves to the 4th ventricle, then over the surface of the brain, where it is absorbed into the blood. As noted by Fishman and Christy (1965) over 50 years ago, intrathecal injection of the drug is equivalent to an IV infusion. An autoradiographic result similar to that shown in Figure 2 was demonstrated following the LV injection of [^125^I]-insulin-like growth factor-1 (IGF1) in the rat (Nagaraja et al., 2005).

A detailed study of monoclonal antibody (MAb) distribution in the brain following ICV administration was reported for the cynomolgus monkey following continuous 24/7 ICV infusion of the MAb in one LV for 42 consecutive days (Yadav et al., 2017). The MAb targeted the beta secretase-1 (BACE1) and was developed as a therapy for AD. The study made several findings:

- The maximal MAb concentration, Cmax, in plasma was 3 μM following ICV infusion and 12 μM following IV infusion. However, the infusion dose (ID) was about 4-fold higher over the course of the study for the IV route as compared to the ICV route. Therefore, a comparable distribution of MAb in plasma was obtained with either the ICV or IV route, thus confirming the observations of Fishman and Christy (1965) that drug injection into the CSF is equivalent to an IV administration.
- The concentration of the MAb in the contralateral motor cortex, which is near the CSF surface, was nearly 30-fold higher than the MAb concentration in a deep parenchyma structure, the contralateral putamen. If the perivascular bulk flow was prominent, there should be little difference in MAb concentration between cortical and subcortical regions of the brain.
- Given a diffusion coefficient of 0.7 × 10^−6^ cm^2^/s, for a large molecule such as a MAb (Pardridge, 2016), the effective diffusion distance over a 6 week period would have a radius of 16 mm and a diameter of 32 mm, and these distances are comparable to the diameter, 40 mm, of a monkey brain (Pardridge, 2016). Thus, continuous 24/7 ICV infusion for 42 days would enable diffusion alone to cover most of the primate brain.
- The distribution of the MAb in the brain was detected with immunocytochemistry, which showed the MAb did not penetrate the white matter of the brain (Yadav et al., 2017). However, perivascular flow in the brain occurs in white matter (Hladky and Barrand, 2014). If the perivascular flow was a prominent mechanism for MAb distribution into brain tissue from the CSF, then MAb should have penetrated into the white matter of the brain.

The minimal distribution of a biologic into human brain following an ICV injection was demonstrated in humans with whole-body positron emission tomography (PET). A [^124^I]-labeled 8H9 MAb was injected into the LV with an Ommaya reservoir of a patient with neuroblastoma metastatic to the meninges (Larson et al., 2015). Whole-body PET scanning at 24 h after the ICV injection showed MAb sequestration by meningeal cancer on the surface of the brain or spinal cord, but no MAb penetration into brain parenchyma. MAb was readily detected in the liver at 24 h following the ICV injection. Uptake of the MAb by the liver following the ICV injection is expected since the injection of a drug into CSF is equivalent to an IV infusion of the drug (Fishman and Christy, 1965).

### CSF Drug Penetration Is Not an Index of Drug Transport Across the BBB

There have been multiple clinical trials in AD with therapeutic antibodies against the Abeta peptide, as exemplified for aducanumab (Sevigny et al., 2016). These antibodies have entered clinical trials with no BBB drug delivery technology. CNS therapeutic antibody-drug developers justified the trials on the basis of the proposal that there is a low level of transport of such antibodies across the BBB, which produces an antibody concentration in the brain that is 0.1%–0.2% of the plasma antibody concentration (Atwal et al., 2011; Bohrmann et al., 2012). However, what is being cited in this context is the ratio of antibody in CSF, not brain, relative to plasma. It is assumed that drug penetration into the CSF is a surrogate for drug penetration across the BBB and into brain parenchyma. This is not the case. Drug distribution into CSF is a function of drug transport across the choroid plexus, which forms the blood-CSF barrier, whereas drug distribution into brain parenchyma is a function of drug transport across the brain capillary endothelium, which forms the BBB (Pardridge, 2016). The BBB and choroid plexus are different membrane barriers, and the choroid plexus is leaky compared to the BBB (Pardridge, 2016; Morris et al., 2017). Prediction on the brain/plasma ratio of therapeutic antibodies should be made on the basis of antibody concentrations in brain tissue, not CSF, following IV administration. The concentration of a therapeutic antibody was measured in primate brain tissue after saline clearance of the brain blood volume, and the average brain antibody concentration was 1 nM after an IV infusion of the anti-BACE1 antibody at an ID of 50 mg/kg (Yadav et al., 2017). However, 1 nM is probably an over-estimate of the MAb concentration in brain after IV infusion, and more likely reflects residual MAb still trapped in the plasma volume of the brain. A 1 nM concentration of anti-BACE1 MAb in the brain should produce a reduction in brain Abeta peptide because the KD of binding of the MAb to BACE1 was 1.3 nM (Yadav et al., 2017). Yet, no reduction of brain Abeta peptide was produced following the IV administration of this large dose of anti-BACE1 MAb. Nevertheless, assuming the brain MAb concentration was 1 nM, this brain MAb concentration was observed when the plasma concentration was at least 8,000 nM, which produces a brain/plasma ratio of 0.01%. This brain/plasma ratio of IgG is >10-fold lower than the CSF/plasma ratio of IgG, 0.1%–0.2%. The higher IgG distribution into CSF, compared to the brain, is expected given the relative leakiness of the choroid plexus, as compared to the BBB (Pardridge, 2016). Owing to the leakiness of the choroid plexus, all proteins in plasma distribute into CSF inversely related to the molecular weight of the protein (Reiber, 2003). Owing to the differential permeability properties of the BBB vs. the blood-CSF barrier, drug entry into CSF should not be used as a surrogate index of drug penetration into brain parenchyma across the BBB.

The fundamental difference between drug transport across the BBB vs. the blood-CSF barrier is illustrated in the case of p-glycoprotein, a transporter that is synonymous with the BBB. Certain drugs that are ligands for p-glycoprotein, e.g., nelfinavir, the HIV protease inhibitor, are excluded from the brain, owing to the expression of the p-glycoprotein active efflux system at the BBB. P-glycoprotein inhibitors, such as zosuquidar, increase brain uptake of nelfinavir in the monkey (Kaddoumi et al., 2007). However, a parallel increase of nelfinavir into CSF is not produced with the co-administration of the p-glycoprotein inhibitor (Kaddoumi et al., 2007). If CSF nelfinavir was monitored after zosuquidar administration, one would erroneously conclude that nelfinavir in the brain was not increased by the P-glycoprotein inhibitor. The lack of an effect on nelfinavir transport into CSF by the P-glycoprotein inhibitor is the expected result because p-glycoprotein is not expressed at the choroid plexus (Matsumoto et al., 2015). P-glycoprotein is a case study for the differential transport of drugs across the blood-CSF barrier vs. the BBB.

## Trans-nasal Drug Delivery to Brain

The hypothesis that drugs are delivered directly to the brain following intranasal administration date back nearly 40 years following the report that the concentration of progesterone, a lipid-soluble small molecule, distributed into CSF of monkeys following intranasal administration (Anand Kumar et al., 1982). About 30 years ago, dextromorphan, also a lipid-soluble small molecule, was detected in brain tissue following the intranasal administration of the drug in small volumes, 5 μl, in rats (Char et al., 1992). Since then, the literature on the transnasal delivery of drugs to the brain has been extended to large molecules, including plasmid DNA for gene therapy (Oviedo et al., 2017; Schuh et al., 2018). There are important principles underlying nasal-to-brain drug delivery:

- Intra-nasal drugs are delivered not to the brain, *per se*, but to CSF within the olfactory region (Kristensson and Olsson, 1971). Therefore, apart from preferential uptake into the local olfactory lobe of the brain, drug delivery to the parenchyma of more distant parts of the brain from CSF is the same whether the drug is given *via* the nasal route or by direct intrathecal injection.
- Drug delivery from the nose to olfactory CSF involves drug transfer across two epithelial barrier membranes in series: the nasal mucosal membrane and the arachnoid membrane (Kristensson and Olsson, 1971). Both barriers, especially the arachnoid membrane, are tight epithelial barriers, and the same rules that apply to BBB transport also apply to delivery across the arachnoid membrane. Lipid soluble small molecules can freely cross such membranes. However, large molecule drugs do not cross epithelial barrier membranes, unless there is membrane injury.
- Intra-nasal injury is caused by the administration of volumes >100 μl/nares in humans (Merkus et al., 2003), which must translate to volumes ≥10 μl/nares in rodents. One finds that in the majority of publications on nose-to-brain delivery, the volumes administered are so large that the test animal must be sedated and then placed in a reverse Trendelenburg position to accommodate the large volume of drug instilled into the nares (Schuh et al., 2018). Local nasal injury is the most likely mechanism by which large molecule drugs gain access to olfactory CSF following nasal instillation.
- When small molecule drugs are administered by the nasal route to humans, but in volumes, 70 μl/nares, which do not cause injury to the nasal mucosa, then no drug is detectable in CSF (Merkus et al., 2003). If local nasal injury due to administration of large volumes in the nose is the mechanism by which drug moves from the nares to the olfactory CSF, then it is unlikely this route of administration can be translated to humans.
- An important factor limiting the translation of nasal delivery in rodents to nasal delivery in humans is the species differences in the olfactory region of the nasal mucosa. The olfactory region is about 50% of the nasal mucosa in rodents, but is only 3%–5% in humans (Graff and Pollack, 2005; Westin et al., 2005).

Despite 40 years of work in trans-nasal drug delivery to the brain, there is not a single biologic drug that is FDA approved for treatment of the human brain following intranasal delivery. This is because nose-to-brain delivery of biologics requires local injury to enable the delivery of large molecule drugs across the nasal and arachnoid membranes.

## Trans-vascular Drug Delivery to Brain Across The Bbb

Rational drug design is based on the understanding of drug interaction with specific receptor targets. Similarly, rational BBB drug delivery for either small molecules or biologics is based on an understanding of the biology of the endogenous transporters expressed at the human BBB *in vivo*. For small-molecule delivery, drugs can be re-engineered to gain access to the brain *via* a number of Carrier-Mediated Transporters (CMT). For large molecule delivery, recombinant proteins and nucleic acid drugs can be re-engineered to enter the brain *via* transport on Receptor-Mediated Transporters (RMT).

### BBB Carrier-Mediated Transporters

**Figure 3:**
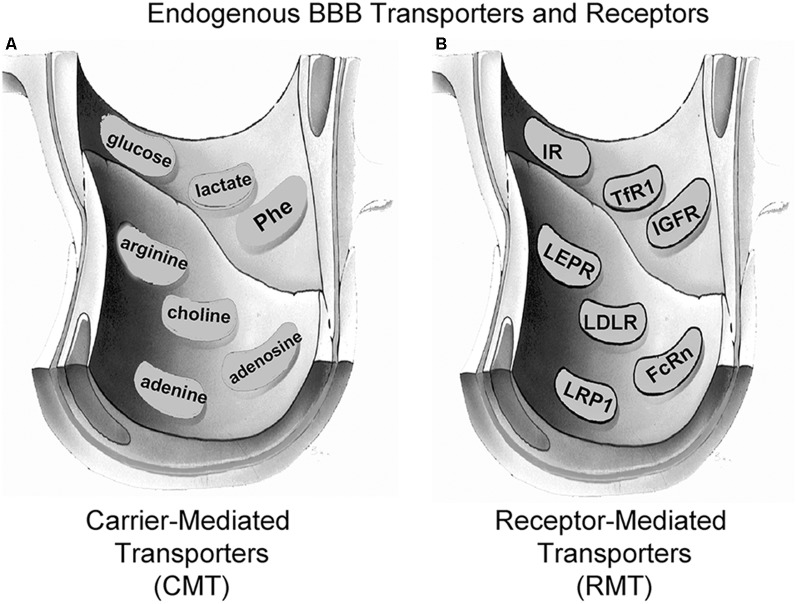
Endogenous BBB transporters include carrier-mediated transport (CMT) systems for certain small-molecule nutrients and receptor-mediated transport (RMT) systems for certain large molecule peptides or plasma proteins. **(A)** CMT systems include different members of the Solute Carrier (SLC) gene family, such as the *GLUT1* glucose transporter for glucose and certain other hexoses, the *MCT1* monocarboxylic acid transporter for lactate, pyruvate, and ketone bodies, the *LAT1* large neutral amino acid transporter for phenylalanine (Phe) and over 10 other neutral amino acids, the *CAT1* cationic amino acid transporter for arginine, lysine, and ornithine, the choline transporter, which may be the choline transporter-like protein-1 (*CTL1*), the *CNT2* sodium-dependent purine nucleoside transporter for adenosine, guanosine, and inosine, and the nucleobase transporter (NBT) for purine bases such as adenine. **(B)** RMT systems include the insulin receptor (*IR*), the type 1 transferrin receptor (*TfR1*), the insulin-like growth factor receptor (*IGFR*), the leptin receptor (*LEPR*), the low-density lipoprotein receptor (*LDLR*), the neonatal Fc receptor (*FcRn*), and the LDLR related protein-1 (*LRP1*). Reprinted by permission from Pardridge (2017).

Small molecule nutrients and vitamins are water-soluble agents that do not significantly cross the BBB *via* free diffusion. Instead, these molecules traverse the BBB *via* transport on specific CMT systems (Pardridge, 2015b). The BBB CMT systems (Figure 3A) include the *GLUT1* glucose transporter, which transports glucose, 2-deoxyglucose, and certain other hexoses; the *LAT1* large neutral amino acid transporter, which transports phenylalanine (Phe) and about 12 other neutral amino acids, particularly the essential neutral amino acids; the *CAT1* cationic amino acid transporter, which transports arginine, lysine, and ornithine; the monocarboxylic acid transporter 1 (*MCT1*), which transports lactate, pyruvate, and ketone bodies; the *CNT2* sodium-dependent nucleoside transporter, which transports purine nucleosides, such as adenosine, and certain pyrimidine nucleosides (uridine); the nucleobase transporter (NBT), which transports purine bases, such as adenine; and the choline transporter, which may be equivalent to the choline transporter-like protein 1 (*CTL1*). LAT1 transports drugs that have a structure that mimics a neutral amino acid, such as L-DOPA, gabapentin, or melphalan (Pardridge, 2015b). CAT1 may be a receptor for certain neurotrophic viruses (Kozak, 2011). The multiple CMT systems at the BBB cover a broad range of molecular structures, and knowledge of the structure-activity relationships of these BBB transporters could guide medicinal chemists in the synthesis of small molecule drugs that penetrate the BBB *via* CMT on these endogenous transporters (Pardridge, 2015b).

### BBB Receptor-Mediated Transporters

Certain large molecule peptides and proteins in plasma normally gain access to the brain *via* receptor-mediated transport (RMT) across the BBB *via* endogenous peptide-specific receptors localized on the plasma membrane of the brain capillary endothelium (Pardridge, 2015a). RMT systems on the BBB (Figure 3B) may mediate the bi-directional transport of peptides in the blood-to-brain or brain-to-blood direction, may mediate only the influx in the blood-to-brain direction, or may mediate only the efflux in the brain-to-blood direction. The BBB insulin receptor (*IR*), which is expressed at the human BBB (Pardridge et al., 1985) mediates the influx of insulin from blood to the brain (Duffy and Pardridge, 1987). Insulin is found in the brain, although the peptide is not synthesized in the brain (Kojima et al., 2004), and brain insulin originates in the pancreatic beta cells followed by secretion to blood, and then RMT across the BBB. The BBB transferrin receptor (*TfR*) is expressed at the human BBB (Pardridge et al., 1987), and mediates both the influx from blood to brain of holo-transferrin (Fishman et al., 1987; Skarlatos et al., 1995) and the efflux of apo-transferrin from brain to blood (Zhang and Pardridge, 2001a). The TfR is expressed on both the luminal and abluminal membranes of the brain capillary endothelium (Huwyler and Pardridge, 1998), and the type of TfR expressed at the BBB is the TfR1 isoform (Li et al., 2001). The insulin-like growth factor (IGF) receptor (*IGFR*) is expressed on the human BBB (Duffy et al., 1988), and IGF1 and IGF2 cross the rat BBB *in vivo* following carotid arterial infusion of plasma-free infusate (Reinhardt and Bondy, 1994). The human BBB IGFR has a high affinity for both IGF1 and IGF2, and affinity cross-linking studies show the IGFR at the human BBB for IGF2 is not the 250 kDa cation-independent M6PR expressed in peripheral tissues (Duffy et al., 1988). The transport of IGF1 or IGF2 into brain *via* the BBB IGFR may be inhibited, because IGF1 and IGF1 are avidly bound, >99%, in plasma by a family of IGF binding proteins (IGFBP; Clemmons, 2018). It is likely that IGFBP complex formation with either IGF1 or IGF2 blocks BBB transport of the peptide, because an IGF2-lysosomal enzyme fusion protein does not cross the BBB following IV administration (Kan et al., 2014). The leptin receptor (*LEPR*) is expressed at the human BBB (Golden et al., 1997), and is believed to mediate the BBB transport of circulating leptin. The principal LEPR isoform expressed at the BBB is the short form (Boado et al., 1998). The low-density lipoprotein (LDL) LDLR is expressed at the BBB (Méresse et al., 1989). However, the activity of this receptor may be low since the transport of LDL-bound cholesterol from the blood to the brain is very slow (Serougne et al., 1976). An Fc receptor (FcR) is expressed at the BBB and mediates the unidirectional efflux from the brain-to-blood of IgG (Zhang and Pardridge, 2001b), and the principal FcR expressed at the BBB is the neonatal FcR or *FcRn* (Schlachetzki et al., 2002). The T1/2 of efflux of IgG from the brain to blood is 48 min (Zhang and Pardridge, 2001b), whereas the T1/2 of efflux of albumin from the brain to blood is about 10–12 h (Cserr et al., 1981). IgG exits the brain rapidly *via* FcR-mediated efflux across the BBB, whereas albumin exits the brain slowly *via* the bulk flow of brain interstitial fluid. The LDL related protein type 1 (*LRP1*) is mainly an endocytosis system (Nazer et al., 2008), which is localized to the abluminal membrane of the brain capillary endothelium (Spuch et al., 2012), and LRP1 ligands may not be effective BBB delivery systems. Melanotransferrin, a ligand for LRP1 (Karkan et al., 2008), is not taken up by brain following IV administration (Richardson and Morgan, 2004).

### Quantitative Targeted Absolute Proteomics of BBB Transporters and Receptors

Terasaki and colleagues pioneered quantitative targeted absolute proteomics (QTAP), which allows for the determination of the quantitative expression of multiple BBB CMT and RMT systems in the isolated brain capillary (Ohtsuki et al., 2011). Brain capillaries are isolated from fresh animal, or human brain, followed by tryptic digests and separation by liquid chromatography-mass spectrometry (LC-MS), in conjunction with peptide standards comprised of an amino acid sequence derived from the known gene sequence of the targeted CMT, RMT, or active efflux transporters (AET). Such studies reveal important species differences. For example, the expression of the TfR1 is about 6- to 8-fold higher than the expression of the IR at the rat or mouse BBB (Agarwal et al., 2012; Hoshi et al., 2013), whereas the IR and TfR1 expression is comparable at the human BBB (Shawahna et al., 2011; Uchida et al., 2011).

The QTAP methodology has been applied to isolated luminal vs. abluminal membranes of the brain capillary, which shows that certain AET systems, such as P-glycoprotein, or the breast cancer resistance protein (BCRP), both members of the ATP-binding cassette (ABC) transporter family, are largely or exclusively localized to the luminal membrane of the BBB (Kubo et al., 2015). Conversely, sodium-dependent neutral amino acid transporters such as ATA2 is localized on the abluminal membrane of the BBB (Kubo et al., 2015).

The relative expression of CMT, RMT, or AET systems at the brain capillary endothelium, which forms the BBB, as compared to the choroid plexus, which forms the blood-CSF barrier, has also been analyzed by QTAP. Whereas P-glycoprotein, or BCRP, are expressed at the BBB, the expression of these AET systems at the choroid plexus is only 0%–2% of the comparative expression at the BBB (Braun et al., 2017). With regard to RMT systems, the expression of the TfR1 at the choroid plexus, in the dog, is 16-fold higher than the expression of the IR at the choroid plexus. The high expression of the TfR1 at the blood-CSF barrier correlates with the rapid distribution of a MAb against the TfR1 into CSF of the primate following IV administration of the TfRMAb (Pardridge et al., 2018).

In addition to the BBB, at the brain capillary endothelium, and the blood-CSF barrier, at the choroid plexus, there is also in brain a blood-arachnoid barrier (BAB) at the arachnoid membrane. The BAB covers the surface of the brain and sits between the subarachnoid space, where CSF flows over the surface of the brain, on one side, and the sub-dural space, on the other side. The arachnoid membrane, or BAB, is formed by arachnoid epithelial cells, which are joined by high resistance tight junctions. Isolation of the leptomeninges, which is comprised of both the arachnoid membrane and the dura mater, in conjunction with the QTAP methodology, has shown that the BAB is the site of expression of CMT and AET systems (Zhang et al., 2018). Certain AET systems, such as P-glycoprotein or BCRP, are not expressed at the choroid plexus, but are expressed at the arachnoid membrane (Zhang et al., 2018). The relative importance of solute and drug transport from CSF to blood across either the choroid plexus, as compared to the arachnoid membrane, is a function of the total surface area of these membrane barriers. The surface area of the human arachnoid membrane is estimated to be about 0.06 M^2^ (Zhang et al., 2018), which is 3-fold greater than early estimates of the surface area of the human choroid plexus, 0.02 M^2^ (Dohrmann, 1970). However, the early measurements of the surface area of the human choroid plexus are under-estimates of the actual surface area (Pardridge, 2016), and do not account for the extensive microvillar amplification of the choroid plexus surface area (Keep and Jones, 1990).

### Receptor-Mediated Brain Delivery of Biologics With Molecular Trojan Horses

Most biologic drugs, e.g., recombinant proteins, therapeutic antibodies, or nucleic acid drugs, are large molecule drugs that do not cross the BBB. However, the discovery of RMT of certain peptides, such as insulin or transferrin, across the BBB *via* the IR or TfR, respectively, led to the concept of delivery of biologic drugs to the brain *via* drug conjugation to ligands of the BBB RMT systems (Pardridge, 1986). In addition to the endogenous ligands, certain peptidomimetic monoclonal antibodies (MAb) that binds an exofacial epitope on the BBB IR or TfR undergoes RMT across the BBB in parallel with the endogenous ligand. The MAb may act as a molecular Trojan horse to ferry into the brain any fused biologic drug that normally does not cross the BBB (Pardridge and Boado, 2012). The preferred MAb Trojan horse binds a site on the BBB receptor that is spatially removed from the binding site of the endogenous ligand. The principal Trojan horse investigated in humans is a MAb against the human insulin receptor (HIR, which is designated the HIRMAb. The HIRMAb cross-reacts with the IR in Old World primates such as the Rhesus monkey but does not cross-react with the IR in New World monkeys or with the IR in rodents (Pardridge et al., 1995; Zhou et al., 2012). The major Trojan horse investigated in rodents is a MAb against the TfR, and specifies specific TfRMAb’s have been developed for rats (Pardridge et al., 1991) and mice (Lee et al., 2000; Boado et al., 2009). The rat-specific TfRMAb does not cross-react with the mouse TfR and does not enter the mouse brain (Lee et al., 2000). The TfRMAb was developed for brain drug delivery in rodents because the expression of the TfR is about 6-fold higher at the rodent BBB as compared to the expression of the IR (Agarwal et al., 2012; Hoshi et al., 2013).

**Figure 4:**
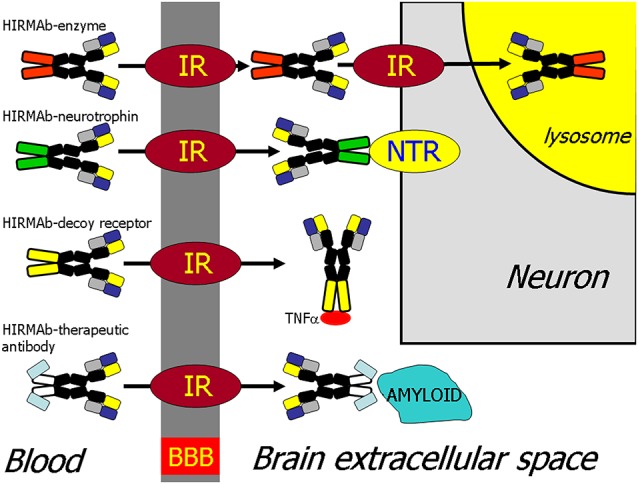
BBB transport of biologic drugs is enabled following the re-engineering of the biologic as an IgG fusion protein. The IgG domain acts as a molecular Trojan horse, and the human insulin receptor (HIR) monoclonal antibody (MAb) is used as the BBB Trojan horse for humans (Giugliani et al., 2018). If the biologic is a lysosomal enzyme, then the drug is re-engineered as a HIRMAb-enzyme fusion protein, which undergoes IR-mediated transport across the BBB, followed by IR-mediated endocytosis into the neuron. If the biologic is a neurotrophin, the drug is re-engineered as a HIRMAb-neurotrophin fusion protein, which undergoes IR-mediated transport across the BBB to enable binding of the neurotrophin domain of the fusion protein to the specific neuronal neurotrophin receptor (NTR). If the biologic is a decoy receptor, such as the extracellular domain (ECD) of the TNFα receptor, the drug is re-engineered as a HIRMAb-decoy receptor, which is transported across the BBB *via* the IR to enable sequestration within brain of TNFα. If the biologic is a therapeutic antibody, e.g., against the Abeta amyloid of AD, then the therapeutic antibody and the HIRMAb are re-engineered as a BBB-penetrating bispecific antibody, which traverses the BBB *via* the IR followed by engagement of the amyloid plaque in brain extracellular spaces. Reprinted by permission from Pardridge (2015a).

The recombinant protein, which does not cross the BBB, can be re-engineered for BBB delivery by genetic fusion of the therapeutic protein to the heavy chain (HC) or light chain (LC) of the MAb Trojan horse (Pardridge and Boado, 2012). Virtually any recombinant protein, which does not cross the BBB, can be re-engineered as a Trojan horse IgG fusion protein that crosses the BBB to exert pharmacologic activity in the brain. In the case of a lysosomal enzyme, such as iduronidase (IDUA), the enzyme mutated in Mucopolysaccharidosis Type I (MPSI), the enzyme was fused to the carboxyl terminus of the HC of the HIRMAb (Boado et al., 2008b), and this HIRMAb-IDUA fusion protein crosses the BBB *via* RMT on the endothelial IR and then undergoes receptor-mediated endocytosis into brain cells *via* the IR expressed on the neuronal cell membrane, followed by triage to the lysosomal compartment (Figure 4). A model neurotrophin, glial-derived neurotrophic factor (GDNF), which is neuroprotective in PD, was fused to the carboxyl terminus of the HC of the HIRMAb (Boado et al., 2008a), and this HIRMAb-GDNF fusion protein traversed the BBB *via* RMT on the IR, followed by binding of the GDNF domain of the fusion protein to the cognate neurotrophin receptor (NTR) on the neuronal cell membrane (Figure 4). A model decoy receptor, the extracellular domain (ECD) of the human tumor necrosis factor receptor type 2 (TNFR2), the active domain of Enbrel^®^, was fused to the carboxyl terminus of the HC of the HIRMAb (Boado et al., 2010b), and this HIRMAb-TNFR2 fusion protein traversed the BBB *via* RMT on the endothelial IR, followed by sequestration of TNFα within brain by the TNFR2 domain of the fusion protein (Figure 4). If the biologic is a therapeutic antibody, then the problem becomes one of engineering a bi-specific antibody (BSA), where both the therapeutic antibody domain and the transporter antibody domain retain high affinity for the respective targets. A BBB-penetrating tetravalent BSA was engineered by first generating a single chain Fv (ScFv) antibody against the Abeta amyloid peptide of AD, followed by fusion of the ScFv to the carboxyl terminus of the HC of the HIRMAb (Boado et al., 2007). The HIRMAb-ScFv fusion protein traversed the BBB *via* RMT on the endothelial IR, followed by binding Abeta amyloid plaque in brain beyond the BBB (Figure 4).

**Figure 5:**
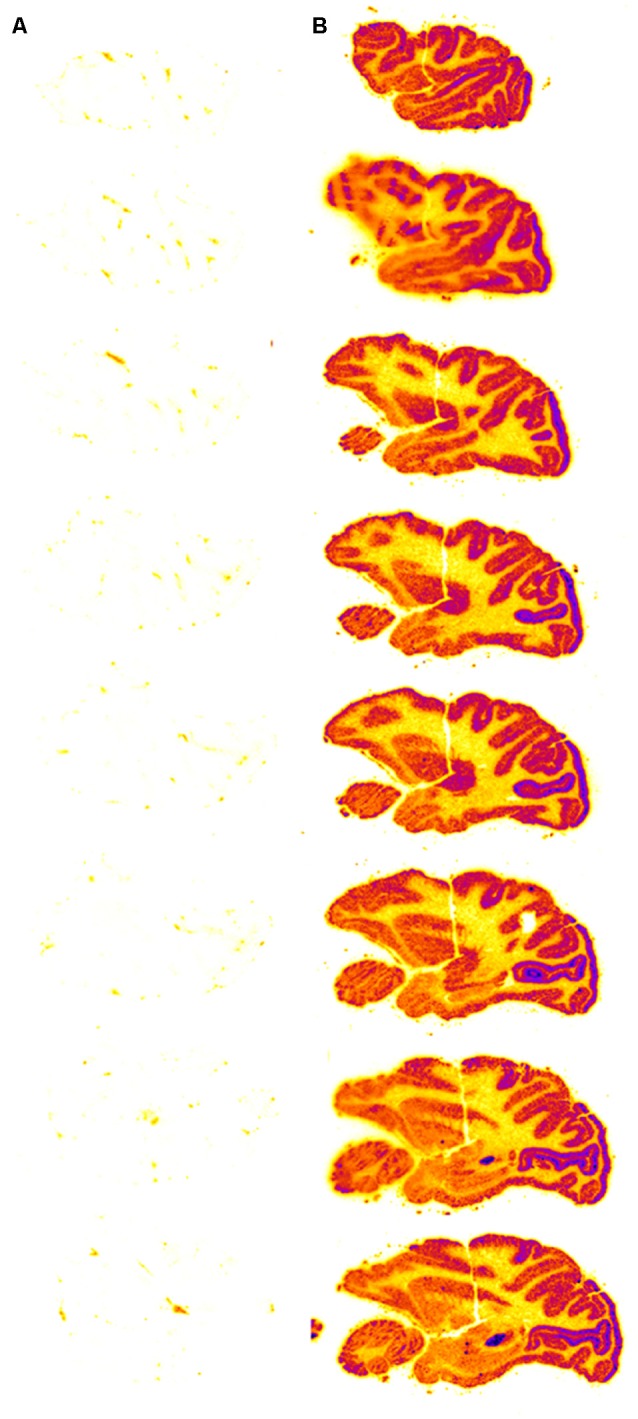
Autoradiography of serial sagittal sections of the Rhesus monkey brain obtained 2 h after the intravenous (IV) administration of either [^125^I]-iduronidase (IDUA), a lysosomal enzyme, or [^125^I]-HIRMAb-IDUA fusion protein. The brain uptake of the IDUA alone is minimal **(A)**, whereas there is robust brain uptake of the HIRMAb-IDUA fusion protein **(B)**. Panels **(A,B)** reprinted by permission from Boado and Pardridge (2017). There is higher uptake of the fusion protein in gray matter, as compared to white matter, owing to the greater vascular density in gray matter (Pardridge et al., 1995).

The impact that this re-engineering of a biologic as an IgG Trojan horse fusion protein has on the brain delivery of the biologic is illustrated in the case of a lysosomal enzyme, IDUA. The recombinant IDUA (laronidase), or the HIRMAb-IDUA fusion protein, was iodinated with the [^125^I]-Bolton-Hunter reagent, and separately administered IV to Rhesus monkeys (Boado and Pardridge, 2017). The brains were removed 2 h after injection, and a series of sagittal sections of the brain were prepared and exposed to a phosphorimager. There is no measurable brain uptake of IDUA alone (Figure 5A) because this enzyme does not cross the BBB. Conversely, there is robust brain uptake of the HIRMAb-IDUA fusion protein (Figure 5B), owing to RMT across the BBB *via* the endothelial IR. The brain uptake of the HIRMAb-IDUA fusion protein is about 1% of injected dose (ID)/brain. This level of brain uptake for the fusion protein, 1% ID/brain, is actually high in that this uptake is comparable to the brain uptake of lipid-soluble small molecules. The brain uptake of morphine and diazepam is only 0.1% ID/brain and 1% ID/brain, in the rat and mouse, respectively (Greenblatt and Sethy, 1990; Wu et al., 1997). Brain uptake of 1% ID/brain of the HIRMAb-IDUA fusion protein generates pharmacologic replacement of IDUA enzyme activity in the brain (Boado et al., 2008b). The HIRMAb-IDUA fusion protein, also designated valanafusp alpha, has been administered to humans with MPSI (Giugliani et al., 2018). MPSI is a lysosomal storage disorder and is an orphan disease of the brain caused by mutations in the gene encoding the IDUA lysosomal enzyme. MPSI patients were treated with valanafusp alpha (HIRMAb-IDUA) by weekly IV infusion for over 1 year. The incidence of mild reversible hypoglycemia or infusion-related reactions was <2%. Valanafusp alpha treatment of MPSI patients, which suffer from severe mental retardation, stabilizes IQ from further decline. The HIRMAb-IDUA fusion protein (valanafusp alpha) is the first BBB molecular Trojan horse to enter human clinical trials and has been shown to stabilize the CNS and to have a favorable safety profile (Giugliani et al., 2018). The success of this trial provides the basis for the future re-engineering of biologic drugs as IgG fusion proteins for non-orphan brain disease such as AD.

## Brain Delivery of Recombinant Proteins in Alzheimer’s Disease

The treatment of AD with biologic drugs requires the re-engineering of these agents to enable RMT across the BBB from the blood. BBB delivery technology is required for AD therapeutics, because the BBB is intact in AD, as determined by multiple experimental approaches including PET (Schlageter et al., 1987), Computed Tomography (Caserta et al., 1998), and Magnetic Resonance Imaging (Starr et al., 2009). The BBB is intact in AD despite the underlying vasculopathy associated with AD, where the majority of neuritic amyloid plaques in AD arise from the brain microvasculature (Miyakawa, 2010).

In addition to re-engineering biologics for BBB transport, AD therapeutic strategies should consider combination therapy. Combination therapy seeks to target the pathogenesis of AD at the multiple sites that lead to dementia. As depicted in Figure 1, AD may be triggered by neuroinflammation that is induced by proinflammatory cytokines such as TNFα. TNFα in the brain may be inhibited by biologic TNFIs that are re-engineered to cross the BBB. A fusion protein of a mouse-specific TfRMAb and the human TNFR2 ECD, which forms the active site of etanercept, was engineered and shown to retain high-affinity binding both for the mouse TfR1, to enable BBB transport, and for human TNFα, to enable sequestration/inactivation of this pro-inflammatory cytokine in brain (Zhou et al., 2011). The chronic administration of the TfRMAb-TNFR fusion protein to double transgenic AD mice caused a reduction in Abeta amyloid plaque, a reduction in markers of neuroinflammation, and improved recognition memory (Chang R. et al., 2017). In parallel with the reversal of neuroinflammation in AD, the therapeutic goal includes disaggregation of Abeta amyloid plaque by a therapeutic antibody that disrupts the plaque. However, AAAs do not cross the intact BBB (Boado et al., 2007), and the BBB is intact in AD (Schlageter et al., 1987; Caserta et al., 1998; Starr et al., 2009). Therefore, an Abeta amyloid antibody (AAA) was re-engineered as a single chain Fv (ScFv) antibody, and this ScFv was fused to the carboxyl terminus of each HC of the HIRMAb (Boado et al., 2007) or the mouse-specific TfRMAb (Boado et al., 2010c). Chronic administration of the TfRMAb-AAA fusion protein to double transgenic AD mice caused a 60% reduction in brain amyloid plaque without causing cerebral micro-hemorrhage (Sumbria et al., 2013). However, both reversal of neuroinflammation in the brain, and amyloid plaque disaggregation, may not result in an improvement in dementia in AD unless there is a reversal of the neurite dystrophy that follows plaque formation. The repair of dystrophic neurites can be accelerated with neurotrophins such as erythropoietin (EPO; Almaguer-Melian et al., 2015). However, EPO does not cross the BBB (Boado et al., 2010a). Therefore, a fusion protein of EPO and the HIRMAb (Boado et al., 2010a) or the mouse-specific TfRMAb (Zhou et al., 2010) were engineered and shown to retain both high-affinity binding to EPO receptor and the respective BBB receptor. Chronic administration of the TfRMAb-EPO fusion protein to double transgenic AD mice resulted in a reversal of synaptic loss and an improvement in spatial memory (Chang et al., 2018). These results show that multiple biologics that intervene at different sites within the disease cascade of AD can be developed as future therapeutics for AD, providing the biologic is re-engineered for BBB transport.

## Brain Delivery of Viral Gene Therapy

In the absence of a BBB drug delivery technology, the drug development of recombinant proteins or therapeutic antibodies as new FDA approvable therapeutics for brain disease has proven to be an intractable problem. This has led to an emergence of brain gene therapy as an alternative platform for CNS drug developers. The leading gene therapies under investigation include hematopoietic stem cells (HSCs) permanently transduced by a retrovirus, or AAV-based gene therapy.

### Brain Retroviral Gene Therapy

Early retroviral gene therapy of severe combined immunodeficiency (SCID)-X1 led to the development of leukemia in multiple patients, which was attributed to insertional mutagenesis caused by the lentivirus (Romano, 2012). More recently, lentiviral gene therapy of the brain has been combined with autologous HSC transplant therapy. The stem cells are removed from the patient and transfected *ex vivo* with lentivirus encoding the therapeutic gene, followed by re-infusion of the transfected stem cells into the patient. This approach is believed to treat the brain based on the hypothesis that stem cells cross the BBB. There are two issues with this approach to the gene therapy of the brain: (a) whether stem cells cross the BBB; and (b) the risk of insertional mutagenesis.

While it is frequently cited that stem cells cross the BBB, the experimental evidence suggests that stem cells undergo minimal, if any, transport across the BBB. Murine HSCs were injected IV in MPS Type VII (MPSVII) mice. While peripheral organs were engrafted with the HSCs, no engraftment of the brain was observed (Soper et al., 2004). The rare HSC detected in the brain was confined to the meningeal surface of the brain, where there is no BBB. While HSC transplant in the MPS VII mouse reversed lysosomal storage disease in peripheral organs, there was no effect in the brain (Soper et al., 2004). HSCs were also injected IV into MPSI mice. While this treatment increased IDUA enzyme activity in peripheral organs, there was no increase in IDUA enzyme activity in the brain (Visigalli et al., 2010). HSC transplant may reverse hydrocephalus in MPSI (Aldenhoven et al., 2015), owing to stem cell engraftment of the meninges through which CSF flows, but there is no direct evidence that HSCs undergo significant penetration of brain parenchyma from the blood in human MPSI. This was further demonstrated in an MPSI mouse treated IV with HSCs transfected with lentivirus encoding the IDUA gene. PCR analysis of the presence of the transgene in mouse organs showed the level of the transgene in the brain was at a background level, approximately 1,000-fold lower than the transgene level in peripheral organs such as liver or thymus (Visigalli et al., 2016). The combination of HSC and lentiviral gene therapy produces a larger increase in lysosomal enzyme replacement in peripheral tissues when compared to HSC transplants alone (Visigalli et al., 2010). HSCs are permanently transfected with lentivirus *ex vivo* so that the HSCs over-produce the transgene, which is the IDUA lysosomal enzyme in the case of treatment of MPSI. The extent to which the HSC is transduced by lentiviral transfection is quantified with the vector copy number (VCN), which is the number of lentiviral genomes inserted into the HSC.

The VCN parameter is important both with respect to efficacy in the brain as well as to toxicity following the administration of lentiviral transfected HSCs. In the MPSI mouse model treated with lentiviral-IDUA transfected HSCs of varying VCN number, the brain IDUA enzyme activity was not increased unless the VCN was ≫5 (mean VCN = 11). If the VCN was <5, then no increase in brain IDUA enzyme activity was observed (Visigalli et al., 2010). These observations should be placed in the context of current FDA restrictions on the VCN in lentiviral gene therapy. The FDA has limited the VCN to <5 per host cell (Zhao et al., 2017), as the risk of insertional mutagenesis is believed to be proportional to the VCN.

Clinical trials with lentiviral transfected HSCs have been reported for the treatment of the brain in metachromatic leukodystrophy (MLD), an orphan disease of the brain caused by mutations in the gene encoding the lysosomal enzyme, arylsulfatase A (ASA). In order to reduce the risk of insertional mutagenesis, the VCN in the MLD patients was about 2 (Biffi et al., 2013). Based on preclinical models (Visigalli et al., 2010), it is not clear if treatment with such a low VCN is sufficient to increase enzyme activity within the brain. The lentiviral-HSC therapy causes a normalization of ASA levels in the CSF (Biffi et al., 2013). However, CSF flows through the meninges, which are outside the BBB and are penetrated by HSCs (Soper et al., 2004). There is no direct evidence that significant increases in ASA enzyme activity within the parenchyma of the brain are produced by infusion of HSCs transfected with a low VCN of 2.

As discussed below for AAV gene therapy of the brain, lentiviral/HSC gene therapy of the brain may have a narrow therapeutic index. In order to treat the brain, the VCN of the HSC must be increased to a copy number ≫5 (Visigalli et al., 2010), which exceeds the FDA VCN limit of 5 (Zhao et al., 2017).

### Brain Adeno-Associated Virus Gene Therapy

Gene therapy with AAV is believed to be safer than lentiviral gene therapy since the AAV functions episomally within the host cell. AAV is believed to be a non-pathogenic virus. However, at high doses, AAV can permanently integrate into the host genome (Nault et al., 2015). Clinical trials of AAV gene therapy restrict the AAV administration to a single dose, and no multi-dose gene therapy with AAV is FDA approved. AAV gene therapy is limited to a 1-time administration of the virus, because the second injection of AAV would likely cause a robust immune response, owing to the immunogenicity of the viral capsid protein (Mingozzi and High, 2013). Early experimental AAV gene therapy of the brain involved direct intracerebral injection of the virus (Passini et al., 2006), and human clinical trials with this type of brain delivery are ongoing for lysosomal storage disease of the brain (Tardieu et al., 2017). The AAV vector encoding the lysosomal enzyme is injected into the human brain *via* eight Burr holes placed in the skull (Tardieu et al., 2017). Despite the injection of the virus into eight Burr holes in the skull, viral distribution to the 1,000-g human brains may be limited, because the virus distribution into the brain following an intracerebral injection is limited to the brain tissue at the tip of the injection needle (Mastakov et al., 2001). In hopes of providing a more uniform distribution of the virus in the brain, preclinical gene therapy of the brain was investigated following the injection of the AAV into the CSF. However, AAV injected into the CSF will have limited diffusion into brain parenchymal owing to the rapid bulk flow of CSF out of the CNS and into the blood. The intrathecal injection of AAV in the primate accounted for transduction of only about 2% of brain cells (Gray et al., 2013). A single injection of AAV into the CSF in monkeys causes an immune response against the AAV in peripheral blood (Samaranch et al., 2012), and this is the expected result, as AAV injected into CSF is expected to move rapidly from CSF to blood similar to other therapeutics. The diameter of AAV is only about 25 nm (Horowitz et al., 2013), whereas particles as large as seven microns can pass from CSF to blood across the arachnoid villi (Pollay, 2010). In parallel with preclinical work on AAV gene therapy of the brain with either intra-cerebral or intrathecal injection, it was discovered that certain AAV serotypes, e.g., AAV9, undergo transvascular transport across the BBB following an IV injection (Foust et al., 2009). As discussed below, the critical factor in transvascular AAV gene therapy of the brain is the injection dose (ID), which is measured as a vector genomes per kg body weight (vg/kg). The ID of AAV is important both with respect to efficacy as well as to the toxicity of intravascular AAV administration.

There are two forms of AAV under investigation: single-stranded (ss) AAV and self-complementary (sc) AAV. A greater number of brain cells are transduced following the IV injection of scAAV9 as compared to ssAAV9 (Gray et al., 2011; Hudry et al., 2018). However, the maximum size of the transgene expression cassette, including the promoter, transgene, and 3′-elements, is <2.3 kb for scAAV, whereas an expression cassette as large as 4.7 kb can be inserted in the ssAAV form (Hudry et al., 2018).

The IV injection of scAAV9 encoding the green fluorescent protein (GFP) was performed in 1-day-old mice at an ID of 10^14^ vg/kg (Foust et al., 2009). This high ID resulted in the transduction of 11%–18% of neurons in the brain of the newborn mouse. Lower levels of transduction were observed in the newborn mouse following the IV injection of ssAAV9 encoding GFP as only about 2% of cells in the brain were GFP positive (Miyake et al., 2011).

MPS Type IIIB (MPSIIIB) is caused by mutations in the gene encoding the lysosomal enzyme, N-acetyl-alpha-glucosaminidase (NAGLU). MPSIIIB mice were injected IV with ssAAV9 encoding NAGLU at a low and a high dose of 5 × 10^12^ and 1.5 × 10^13^ vg/kg, respectively (Fu et al., 2011). AAV is a hepatotropic virus, and this treatment caused high expression of essentially all cells in the liver. However, only 7%–9% of brain cells were transduced at these injection doses (Fu et al., 2011). Subsequently, the ssAAV9-NAGLU was injected IV in primates at a dose of 2 × 10^13^ vg/kg, and this dose transduced 4%–24% of cells in the brain (Murrey et al., 2014). This primate study is important with respect to the immunologic aspects of AAV gene therapy of the brain. The AAV anti-drug antibody (ADA) titer increased as high as 10,000-fold in plasma following the single IV treatment with AAV in the monkey. The ADA response was directed not only at the AAV capsid protein, but also at the protein product, the NAGLU enzyme, of the transgene. The ADA titer included neutralizing antibodies (NAb) against NAGLU, and these NAb’s caused a depletion of endogenous NAGLU in the normal primates (Murrey et al., 2014). A similar immune response against the transgene product was observed in infant Rhesus monkeys administered AAV encoding the lysosomal enzyme, iduronidase (IDUA), *via* an occipital intrathecal injection (Hordeaux et al., 2019). An immune response against the therapeutic protein could ultimately negate the therapeutic effect of the AAV gene therapy.

The above studies indicate that the BBB transport of AAV9 is not efficient and high doses of 10^14^ vg/kg must be injected in order to transduce up to ~20% of brain cells. The efficiency of AAV9 transport across the BBB might be increased by genetic modification of the capsid protein to increase the affinity of the capsid protein for the putative BBB receptor that mediates the uptake from blood of AAV9. One such capsid variant is designated Anc80L65. Following the IV injection of 4 × 10^13^ vg/kg in the mouse, the number of neurons and astrocytes that were transduced with ssAAV9 was 2% and 5%, respectively; the number of neurons and astrocytes that were transduced with ssAAV9-Anc80L65 was 7% and 25%, respectively (Hudry et al., 2018). Even with the Anc80L65 variant, the number of neurons transduced by IV administration of the virus is only 7% of the neurons in the brain.

AAV gene therapy of the spinal cord is now FDA approved for the treatment of infantile SMA with a single IV dose of 2 × 10^14^ vg/kg of the scAAV9 encoding the SMN1 gene (Mendell et al., 2017), and this gene therapeutic is designated scAAV9.CB.hSMN (Zolgensma^®^). The FDA approved IV dose of Zolgensma, 10^14^ vg/kg, is high, and this dose is associated with toxicity in juvenile primates, including elevations of liver enzymes, liver failure, degeneration of dorsal root ganglia, proprioceptive deficits, and ataxia (Hinderer et al., 2018).

Zolgensma IV AAV gene therapy is approved for a 1-time treatment, because subsequent doses of AAV may cause a potentially severe immune reaction due to the immunogenicity of the AAV capsid protein (Mingozzi and High, 2013; Fitzpatrick et al., 2018; Wang et al., 2018). While lentiviral gene therapy is permanent, is it difficult to see how an episomal form of gene therapy, such as with AAV, can be life-long, unless the AAV permanently integrates into the host genome. Expression of the AAV genome in primates for as long as 4 years have been observed (Hordeaux et al., 2019).

The idea that AAV is a non-pathogenic virus is coming under scrutiny, as recent work shows AAV is potentially oncogenic owing to insertional mutagenesis, particularly in the liver (Rosas et al., 2012; Nault et al., 2015; La Bella et al., 2019). The organ most affected by AAV is the liver, owing to the very high uptake of AAV by the liver. Long term (2 year) studies in mice show a 50%–75% incidence of hepatocellular carcinoma (HCC) in newborn mice treated with a single IV injection dose (ID) of 10^14^ vg/kg of AAV (Chandler et al., 2015). This ID of 10^14^ vg/kg that produces liver cancer in mice is the same dose used to treat human SMA (Mendell et al., 2017).

AAV gene therapy of the brain may prove to have a narrow therapeutic index. The ID, e.g., ≥10^14^ vg/kg, that is necessary to transduce at least a minor fraction of neurons in the brain or spinal cord, is also the dose that may cause a delayed development of liver cancer. Given these safety factors, it is important to develop non-viral forms of gene therapy of the brain.

## Brain Delivery of Non-viral Gene Therapy

Non-viral gene delivery originated over 30 years ago. Felgner et al. (1987) developed lipofection of cultured cells by the combination of cationic polymers (cationic lipids) and anionic polymers (DNA), a mixture called polyplexes. The initial polyplex was formed by mixing anionic plasmid DNA with a cationic lipid, DOTMA N-[1-(2,3,-dioleyloxy)propyl]-N,N,N-trimethylammonium chloride and a helper lipid, dioleoyl phosphatidylethanolamine (DOPE), a mixture also known as Lipofectin^®^. The cationic lipid was modified to produce Lipofectamine^®^, a reagent still widely used for the transfection of cultured cells. In the intervening 30+ years, no non-viral polyplex gene therapy has been approved by the FDA. As discussed below, the challenges in the development of effective and safe non-viral polyplex gene delivery to the brain have been every bit as great as the challenges discussed above for viral gene therapy of the brain. In parallel to the development of polyplex gene therapy, other forms of non-viral gene delivery were developed, and the non-viral gene delivery systems may be broadly classified among four groups: (a) mixture of anionic DNA and cationic polymers (polyplexes); (b) pegylated liposomes encapsulating plasmid DNA, also known as stabilized plasmid lipid particles (SPLP) or lipid nanoparticles (LNP); (c) hydrodynamics gene delivery (HGD); and (d) receptor-mediated Trojan horse liposomes (THLs).

### Plasmid DNA Polyplexes

Anionic DNA and cationic polymers, when mixed in the proper molar ratio, will form ~100 nm nanoparticles when formulated in low ionic strength solution, e.g., 0.01 M Tris-buffered water. The different polymers that have been used include cationic lipids, such as variants of DOTMA, cationic polymers such as polyethyleneimine (PEI; Zou et al., 2000) or poly(β-amino ester; PBAE; Mangraviti et al., 2015), cationic proteins, such as poly-L-lysine (PLL; Ward et al., 2001), cationic linear polysaccharides, such as chitosan (Baghdan et al., 2018), or cationic dendrimers (Mai et al., 2015), which are synthetic branched or treelike molecules. The DNA polyplexes have the following properties:

- The ~100 nm nanoparticles formulated in water rapidly aggregate when placed in physiological saline into structures with a size of >1 micron (Plank et al., 1999). This aggregation property is useful for transfection of cultured cells, as the aggregation into particles >1 micron in size triggers cellular uptake *via* phagocytosis (Akinc and Battaglia, 2013).
- The aggregation of DNA polyplexes occurs *in vivo* following IV injection, which leads to entrapment of the aggregates in the first vascular bed encountered after an IV injection, which is the lung (Hofland et al., 1997). Transfection of the endothelium in the lung is triggered by what is effectively a pulmonary embolism.
- The structure of the anionic plasmid DNA and the cationic polymer is a multilamellar structure with the alternating lipid bilayer and DNA monolayers (Radler et al., 1997). Owing to this structure, the plasmid DNA is susceptible to endonucleases, which leads to nuclease degradation of the plasmid DNA (Simberg et al., 2001).
- Plasmid DNA polyplexes do not cross the BBB following IV administration. Therefore, gene therapy of the brain with this approach requires direct intracerebral injection into the brain (Mangraviti et al., 2015). However, intracerebral injection of a gene medicine only treats the brain tissue at the tip of the injection needle (Mastakov et al., 2001). Diffusion through the brain is limited as diffusion decreases with the square of the distance and decreases with the molecular size of the drug.
- Plasmid DNA polyplexes trigger inflammatory responses *in vivo* following IV administration, which is attributed primarily to the naked DNA, rather than the cationic lipid (Norman et al., 2000). Removal of CpG motifs from the plasmid DNA reduces several, but not all, proinflammatory properties of naked DNA *in vivo* (Yew and Scheule, 2005).

Polyplex gene therapy of the retina has been developed. The plasmid DNA was bound to PLL to form the polyplex, and the PLL was conjugated with 30 kDa polyethylene glycol (PEG). Following IV administration of the polyplex, the mixture would not be expected to significantly penetrate the eye or other organs of the body *in vivo*, other than the lung. Therefore, treatment of the eye required a sub-retinal injection of the polyplex (Kelley et al., 2018). Similarly, AAV-based gene therapy of retinal disease is performed with a 1-time sub-retinal injection in each eye (Maguire et al., 2019).

### Lipid Nanoparticles

LNP are typically pegylated liposomes that encapsulate the plasmid DNA in the interior of the ~100 nm liposome. Any non-encapsulated plasmid DNA is removed by anion exchange chromatography or filtration through anion-exchange filters. LNPs were formed with 82.5% helper neutral lipid, dioleoylphosphatidylethanolamine (DOPE), 7.5% cationic lipid, N-N-dioleoyl-N,N-dimethylammonium chloride (DODAC), and 10% pegylated lipid (Ambegia et al., 2005). Since the pegylated lipid is anionic and is in excess of the DODAC, the pegylated liposome has a net anionic charge. The plasmid DNA encoding the luciferase reporter gene was encapsulated in the interior of the LNPs with the dialysis detergent method of liposome formation, and the external DNA was removed by anion exchange chromatography. The LNPs were injected IV in the 20-g mouse at a plasmid DNA dose of 100 μg/mouse (Ambegia et al., 2005), which is an injection dose (ID) of 5,000 μg/kg. The LNPs were avidly taken up by peripheral organs as the organ uptake in spleen, liver, and lung was 50% ID/g, 25% ID/g, and 10% ID/g, respectively. The greater uptake in liver and spleen, as compared to lung, indicates the LNPs do not aggregate *in vivo*, as the DNA is encapsulated in the interior of the LNP. Despite the high organ uptake in liver, spleen, and lung, the expression of the luciferase reporter gene was low, e.g., 2, 1, and 0.5 pg/g organ for spleen, liver, and lung, respectively (Ambegia et al., 2005). Assuming 100 mg protein per gram tissue, the luciferase expression is only 0.02, 0.01, and 0.005 pg/mg protein, for spleen, liver, and lung, respectively. The high organ uptake, yet the low expression of the luciferase transgene, following the administration of LNPs (Ambegia et al., 2005) suggests that >99% of the plasmid DNA is not expressed *in vivo*, and may be rapidly degraded within the lysosome compartment. Pegylated liposomes, or LNPs, are not taken up by the brain *in vivo* following IV administration (Huwyler et al., 1996). As discussed below, LNPs need to be modified with receptor targeting ligands that trigger receptor-mediated uptake of the LNP *in vivo*.

### Hydrodynamic Gene Delivery

HGD is a form of non-viral delivery of naked plasmid DNA to the liver (Hodges and Scheule, 2003). HGD involves the IV injection of naked plasmid DNA in a large volume, 100 ml/kg, of saline over a short period of time, 3–5 s. In a 20 g mouse, the injection volume would be 2 ml, which exceeds the entire blood volume of the mouse. This procedure causes sudden cardiac congestion, which results in a rapid backflow of blood to the inferior vena cava (IVC) and hepatic veins. The injection solution rapidly expands the liver to cause hepatic injury, which enables the plasmid DNA to penetrate the liver cells. The liver injury is manifested by large increases in the blood of liver transaminase enzymes. The procedure is said to be safe, but the rapid IV injection of volumes used in this procedure, 100 ml/kg, is 10-fold greater than the usual maximum rapid IV injection volume allowed in vertebrate animal research. In an attempt to adapt the HGD method to humans, the sudden increase in hepatic pressure was induced with image-guided balloon catheters under fluoroscopy (Eastman et al., 2002). The HGD method is not applicable for gene delivery to the brain. HGD has a narrow therapeutic index, in that the volume of saline, which is required to cause the tissue injury that enables uptake of plasmid DNA, is close to the volume of saline that induces acute changes in cardiac hydrodynamics.

## Non-viral Gene Therapy of the Brain With Trojan Horse Liposomes

### Receptor-Targeting Trojan Horse Liposomes Encapsulated With Plasmid DNA

The first attempt at receptor-mediated plasmid DNA delivery *in vivo* was reported over 30 years ago for liver delivery of pSV2-cat, a chloramphenicol acetyltransferase (CAT) reporter plasmid DNA (Wu and Wu, 1988). The anionic pSV2-cat plasmid DNA was electrostatically attached to a cationic polymer, 59 kDa PLL. The PLL was conjugated to asialoorosomucoid (AsOR), a glycoprotein formed by treatment of orosomucoid (OR) with neuraminidase to remove sialic acid residues on the OR plasma protein. Liver cells over-express an asialoglycoprotein receptor (*ASGR*), which avidly extracts from blood certain plasma glycoproteins that have been treated to remove the sialic acid moiety (Pardridge et al., 1983). The IV injection in the rat of 1.0 mg of pSV2-cat reporter plasmid complexed to PLL-asialoorosomucoid resulted in CAT gene expression in the liver. The ID in this study is high, about 5,000 μg DNA/kg body weight. The extent to which the plasmid DNA dissociates *in vivo* from the PLL, prior to hepatic uptake, is not known. The expression of the *ASGR* is generally restricted to the liver, and this receptor is not expressed at the BBB. Other receptors, such as the *IR* or *TfR*, are expressed at the BBB (Figure 3) and could be used to target plasmid DNA to the brain. However, the use of a PLL or other cationic linker that joins the naked plasmid DNA and the targeting ligand is problematic, because the naked DNA is subject to rapid degradation *in vivo* by ubiquitous endonucleases. An alternative approach is to encapsulate the plasmid DNA in the interior of a liposome or LNP, which is then targeted to the brain with a receptor-specific MAb on the surface of the liposome, a formulation designated as Trojan horse liposomes (THL).

**Figure 6:**
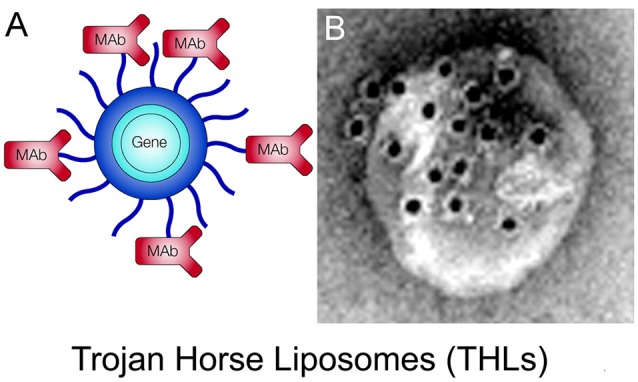
**(A)** Structure of a Trojan horse liposome (THL). A single plasmid DNA molecule is encapsulated in the interior of a ~100 nm liposome, the surface of which is conjugated with several thousand strands of 2,000 Da polyethylene glycol (PEG). The tips of 1%–2% of the PEG strands are conjugated with a receptor-specific monoclonal antibody (MAb). The MAb against either the IR or the transferrin receptor (TfR) engages the IR or TfR on the BBB to mediate transport into the brain and then binds the IR or TfR on the brain cells to trigger receptor-mediated endocytosis into brain cells. Panel **(A)** reprinted by permission from Pardridge (2002). **(B)** Electron micrograph of a THL complexed with a conjugate of a secondary antibody and 10 nm gold. The gold particles are about the same size as the PEG-extended MAb on the surface of the THL. Panel **(B)** reprinted by permission from Zhang et al. (2003a).

THLs, also called pegylated immunoliposomes (PILs), are ~100 nm diameter liposomes with a surface covered by several thousand strands of 2,000 Da polyethyleneglycol (PEG^2000^; Huwyler et al., 1996; Shi and Pardridge, 2000). The tips of 1%–2% of the PEG strands are conjugated through a thioether linker with a receptor-specific monoclonal antibody (MAb). The same MAb’s against the IR or TfR used to re-engineer recombinant proteins (Figure 4) are also used as Trojan horses to deliver the THLs across the BBB. For delivery in humans or Old World primates, such as the Rhesus monkey, the HIRMAb is used. For delivery in rodents, the rat 8D3 MAb against the mouse TfR1 is used for THL delivery in mice, and the murine OX26 MAb against the rat TfR1 is used for THL delivery in rats. The phospholipids are comprised of 1-palmitoyl-2-oleoyl-*sn*-glycerol-3-phosphocholine (POPC), which is one of the most abundant phospholipids in mammalian membranes, dimethyldioctadecylammonium bromide (DDAB), which is a cationic lipid, and distearoylphosphatidylethanolamine (DSPE)-PEG^2000^, which covers the liposome surface with a PEG corona (Pardridge, 2003). A certain fraction of the (DSPE)-PEG^2000^ incorporates a maleimide (MAL) moiety for MAb conjugation. The MAb is thiolated with Traut’s reagent to enable conjugation to the MAL moiety on the surface of the THL (Huwyler et al., 1996). Prior to MAb conjugation, the liposomes are formed with the thin film/extrusion method and encapsulate the plasmid DNA in the interior of the liposome. Any plasmid DNA not encapsulated inside the THL is removed by endonuclease treatment. A THL encapsulated with a plasmid DNA is shown in Figure 6A. An electron micrograph of a THL is shown in Figure 6B. The THL was bound by a conjugate of a secondary antibody and 10 nm gold. The 10 nm gold particles are about the same size as the MAb and show the spatial orientation of the MAb on the surface of the liposome, where the MAb is attached to the end of the 2,000 Da PEG, which is comprised of 45-(OCH_2_-CH_2_) groups and has a length of 16 nm. There are about 40–80 MAb molecules conjugated per THL, and a single plasmid DNA is encapsulated in a THL. Once the plasmid DNA is encapsulated in the THL, the DNA is resistant to degradation by external nucleases (Pardridge, 2003).

### Brain Delivery of Reporter Genes With THLs

**Figure 7:**
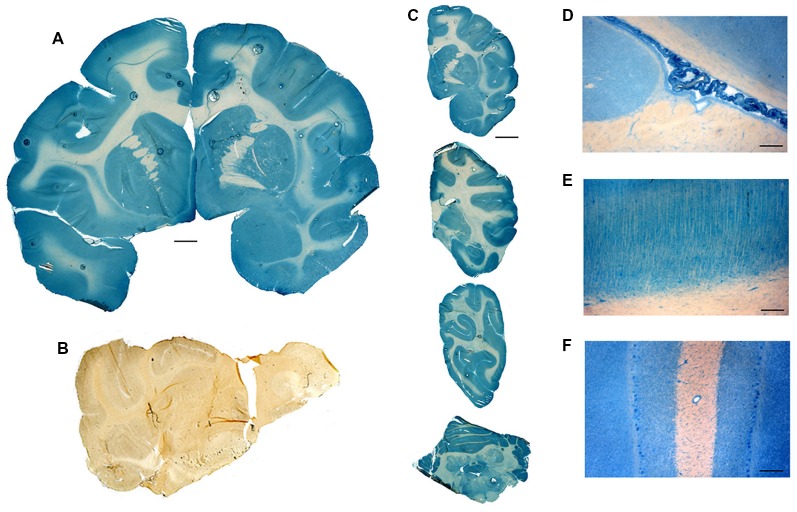
**(A)** Coronal section of Rhesus monkey brain after beta-galactosidase (*LacZ*) histochemistry. The primate brain was removed 2 days after the IV administration of a HIRMAb-targeted THL encapsulating a *LacZ* expression plasmid DNA. Magnification bar = 3 mm. **(B)** LacZ histochemistry of Rhesus monkey brain not injected with THLs. **(C)** Serial sections through the primate brain show the global distribution of the transgene in all parts of the primate brain following THL administration of the LacZ gene. Light microscopy of choroid plexus **(D)**, occipital cortex **(E)**, and cerebellum **(F)** after THL administration of the *LacZ* gene. Panels **(A–F)** reprinted by permission from Zhang et al. (2003c).

A plasmid DNA encoding the bacterial β-galactosidase *LacZ* gene was encapsulated in HIRMAb-targeted THLs and injected IV in the adult Rhesus monkey, which was euthanized at 48 h (Zhang et al., 2003c). The plasmid DNA injection dose (ID) in this study was 12 μg/kg, which corresponds to an ID of 10^12^ vg/kg. This ID is 2 log orders of magnitude lower than the AAV9 ID used in IV viral gene therapy. Following IV injection of the THL in the primate, the brain was removed, sectioned and stained with X-Gal histochemistry to determine the extent of the transgene expression in the monkey brain. As shown in Figure 7A, there is a global expression of the transgene throughout the monkey brain. The control monkey brain not treated with THLs produced the X-Gal image in Figure 7B. The different sections of the primate brain shown in Figure 7C indicate that transgene is expressed in all regions of the brain. Light microscopy is shown in Figure 7D (choroid plexus), Figure 7E (occipital cortex), and Figure 7F (cerebellum). Gene expression is visible within the choroid plexus epithelium, the ependymal lining of the ventricle, and the capillary endothelium of adjacent white matter (Figure 7D). The transgene expression within the neurons of the occipital cortex is visible and reveals the columnar organization of the occipital cortex of the primate brain (Figure 7E). The LacZ transgene is expressed in the molecular and granular layers of the cerebellum, as well as the intermediate Purkinje cells (Figure 7F).

Examination of the ocular structures of the primate showed global expression of the β-galactosidase transgene throughout the retina, as well as brain, liver, and spleen (Zhang et al., 2003b). In these studies, the β-galactosidase transgene was under the influence of the widely expressed SV40 promoter. When the SV40 promoter was replaced by a tissue-specific gene promoter taken from the 5′-flanking sequence (FS) of the eye-specific opsin gene, then β-galactosidase transgene expression in the monkey was only observed in the eye, and no expression in brain, liver, or spleen was observed (Zhang et al., 2003b). Therefore, the combination of the THL plasmid DNA delivery system, and tissue-specific gene promoters, enables restriction of the transgene expression to the target organ in the body. Whereas there is a size limitation of 2–4 kb of a transgene that can be inserted in the AAV genome, plasmid DNAs as large as 22 kb have been encapsulated in THLs followed by gene expression in the brain *in vivo* (Xia et al., 2007).

Monkeys were also injected IV with HIRMAb-targeted THLs encapsulating a luciferase reporter gene (Zhang et al., 2003c). PCR was used to measure the number of plasmid DNA molecules in the monkey brain (Chu et al., 2006). Following the IV administration of 12 μg/kg of a 10.6 kb luciferase plasmid DNA, the brain uptake of the luciferase plasmid was 798 ± 123 fg luciferase DNA per 200 ng genomic DNA. Assuming 10 pg genomic DNA per brain cell, these data indicate that an average of 3.3 plasmid DNA molecules was delivered to every cell in the primate brain (Chu et al., 2006). These findings correspond to the LacZ histochemistry, which shows global expression of the transgene in all parts of the primate brain (Figure 7). Plasmid DNA expression is transient because the plasmid DNA does not integrate with the host genome. The T1/2 of luciferase enzyme activity in the primate brain was 2.1 ± 0.1 days, which correlated with the T1/2 of the plasmid DNA in the brain, 1.3 ± 0.3 days (Chu et al., 2006). The persistence of the transgene protein product in the brain cell is a function of the T1/2 of turnover of both the plasmid DNA and the protein product of the transgene. In murine fibroblasts transfected with TfRMAb-targeted THLs, encapsulated with a β-glucuronidase (GUSB) expression plasmid, the T1/2 of GUSB enzyme activity in the cell following a single treatment with THLs was greater than 2 weeks (Zhang et al., 2008).

### Safety of THL Gene Therapy of Brain

THL gene therapy with plasmid DNA is reversible, which is considered a safety advantage for first-generation gene medicines. Therefore, THLs are administered weekly similar to weekly IV Enzyme Replacement Therapy (ERT) for the treatment of lysosomal storage disorders with recombinant lysosomal enzymes. The HIRMAb has been administered to humans with MPSI for over a year with a favorable safety profile and infusion-related reactions were <2% (Giugliani et al., 2018). TfRMAb-targeted THLs encapsulating a tyrosine hydroxylase (TH) expression plasmid was administered chronically to rats by weekly IV injections, and no immune reactions were observed (Zhang Y. F. et al., 2003). There was no change in body weight, 14 serum chemistries, or histology in the brain or peripheral organs. Immunocytochemistry of the brain showed no neuro-inflammation. In the future, when it is possible to stably integrate into the host genome without risk of insertional mutagenesis, THLs can be used for 1-time gene therapy with chromosomal integration of the host genome. The plasmid DNA can be engineered with one expression cassette for the therapeutic gene that is flanked with inverted terminal repeats (ITR), and a second expression cassette, which expresses an ITR-related recombinase, that is placed in tandem with the therapeutic gene expression cassette.

### THL Gene Therapy of Parkinson’s Disease

The history of new drug development for PD is every bit as dismal as for AD. No biologic drugs are currently approved by the FDA for PD because these large molecule drugs do not cross the BBB. Patients with PD could benefit from BBB-penetrating biologics. Similar to AD, patients with PD could benefit from a triad of biologics that include: (a) biologic TNFIs that suppress the neuroinflammation that occurs in brain in PD (McCoy et al., 2006); (b) therapeutic antibodies that block the formation of α-synuclein aggregates in PD (Schofield et al., 2019); and (c) neurotrophins that induce repair of dystrophic neurites in PD. However, similar to AD, all three classes of these biologic agents need to be re-engineered for BBB delivery before successful clinical trials in PD could be expected. Owing to the lack of BBB drug delivery technology within the pharmaceutical industry, there has been no biologic drug FDA approved for PD. The most potent neurotrophic factor for PD is GDNF (Ibáñez and Andressoo, 2017), and clinical trials of trans-cranial delivery of GDNF *via* ICV injection (Nutt et al., 2003) or convection enhanced diffusion (CED; Lang et al., 2006) have been attempted. As expected, both clinical trials failed. In the case of ICV injection, this route of drug delivery to the brain only results in neurotrophin distribution to the ependymal surface ipsilateral to the injection (Figure 2). CED is an ineffective means of drug delivery to the brain parenchyma because drug distributes into the brain *via* diffusion, not convection (Salvatore et al., 2006).

Gene therapy of PD is aimed at either replacement of tyrosine hydroxylase (TH) enzyme activity in the nigrostriatal tract, or nigrostriatal regeneration with GDNF gene therapy. Both TH and GDNF gene therapy have been evaluated in experimental PD in the rat with the THL plasmid DNA delivery technology. Experimental PD in the rat was produced by the unilateral stereotactic injection of a moderate dose, 8 μg, of the neurotoxin, 6-hydroxydopamine, in the median forebrain bundle (MFB) on one side of the brain (Zhang et al., 2003a). A rat TH expression plasmid DNA was engineered where the rat TH cDNA was under the influence of the SV40 promoter, and the 3′-untranslated region (UTR) contained a 200 nucleotide (nt) stabilizing sequence taken from the 3′-UTR of the GLUT1 glucose transporter mRNA. The TH expression plasmid was encapsulated within THLs that were targeted with either the murine OX26 MAb against the rat TfR or the mouse IgG2a isotype control antibody. At 3 weeks after the single injection of the neurotoxin, motor dysfunction was evaluated by apomorphine-induced rotation behavior (Zhang et al., 2003a). Rats that demonstrated >120 rotations per 20 min in a direction contralateral to the toxin injection where shown to have experimental PD. These rats were then treated with a single IV injection of TfRMAb-targeted THLs encapsulating the TH plasmid DNA at a dose of 1–10 μg plasmid DNA per 200-g rat. In untreated animals, the neurotoxin lesion caused an 85% reduction in striatal TH enzyme activity. The striatal TH enzyme activity was normalized at 3 days after the IV injection of 10 μg/rat of the TH plasmid DNA packaged in the TfRMAb-targeted THLs. In contrast, the IV injection of the TH plasmid DNA packaged in THLs targeted with the mouse IgG2a isotype control antibody had no therapeutic effect. A dose-response relationship was demonstrated as 1 μg/rat and 5 μg/rat doses produced sub-therapeutic effects on brain TH expression. A time-response relationship was also observed as the striatal TH enzyme activity, which was normalized at 3 days after THL administration, declined with a T1/2 of 3 days. Since the TH expression plasmid was driven by the widely expressed SV40 promoter, off-target effects were observed as TfRMAb-targeted THLs caused a 35-fold increase in TH enzyme activity in the liver (Zhang et al., 2003a).

**Figure 8:**
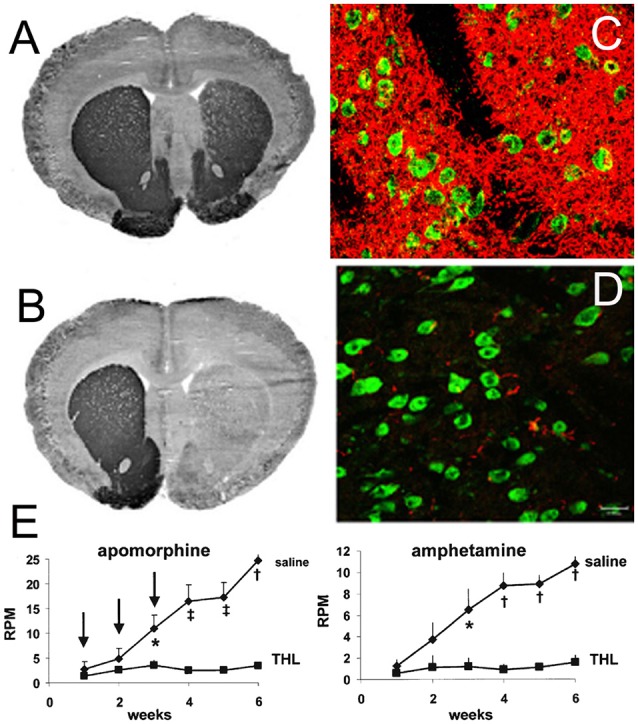
**(A)** Tyrosine hydroxylase (TH) immunohistochemistry (IHC) of rat brain removed 3 days after the IV administration of TfRMAb-targeted THLs encapsulating a rat TH expression plasmid DNA, under the influence of a human glial fibrillary acidic protein (GFAP) promoter, and 3.5 weeks after the unilateral injection of 8 μg of 6-hydroxydopamine into the right medial forebrain bundle. The injection dose of plasmid DNA was 10 μg/rat. **(B)** TH IHC of rat brain removed 3 days after the IV administration of THLs encapsulating the GFAP-TH transgene, but targeted only with a mouse IgG2a isotype control antibody with no specificity for a BBB receptor, and 3.5 weeks after the unilateral injection of 8 μg of 6-hydroxydopamine into the right medial forebrain bundle. There is a >90% loss of immunoreactive TH in the caudate-putamen nucleus (CPN) ipsilateral to toxin injection, and this loss is completely restored in the rats treated with the THL targeted with the TfRMAb (panel **A**), but not with the mouse IgG2a isotype control (panel **B**). **(C)** Confocal microscopy of the CPN region of the brain corresponding to panel **(A)**, and TH immunostaining is shown in the red channel and immunostaining of neuN, a neuronal marker, is shown in the green channel. **(D)** Confocal microscopy of the CPN region of the brain corresponding to panel **(B)**, and TH immunostaining is shown in the red channel and immunostaining of neuN is shown in the green channel. Magnification bar in panel **(D)** is 20 μm. Panels **(A–D)** reprinted by permission from Zhang et al. (2004a). **(E)** Apomorphine (left panel) and amphetamine (right panel)-induced rotation behavior in rats at 1 through 6 weeks after the administration of 8 μg of 6-hydroxydopamine in the right medial forebrain bundle. The rats were treated at weeks 1, 2, and 3 after intra-cerebral toxin administration with either saline or with TfRMAb-targeted THLs encapsulating a human prepro glial-derived neurotrophic factor (GDNF) transgene. The GDNF gene was under the influence of an 8 kb tissue-specific promoter taken from the 5′-flanking sequence of the rat TH gene. The differences in rotation in the THL and saline-treated rats are statistically significant at weeks 3, 4, 5, and 6 (*p < 0.05, ^‡^*p* < 0.005, ^†^*p* < 0.0005). Panel **(E)** reprinted by permission from Zhang and Pardridge (2009).

Off-target effects of THL gene therapy are eliminated with the use of tissue-specific gene promoters (Zhang et al., 2003b). The TH expression plasmid was re-engineered where the SV40 promoter was replaced by a brain-specific promoter taken from the 2 kb of the 5′-flanking sequence (FS) of the human glial fibrillary acidic protein (GFAP) gene (Zhang et al., 2004a). The IV injection of 10 μg/rat of the TfRMAb-targeted THL carrying the GFAP-TH expression plasmid caused a complete normalization of TH expression in the striatum ipsilateral to the toxin injection (Figure 8A), and confocal microscopy of the striatum demonstrated robust TH expression in neurons identified with neuN immune-staining (Figure 8C). In contrast, there was no replacement of striatal TH in the lesioned rat brain when the GFAP-TH expression plasmid was encapsulated in THLs targeted with the mouse IgG2a isotype control antibody as shown by either immunocytochemistry (Figure 8B) or confocal microscopy (Figure 8D). An improvement in aberrant motor activity was also observed. The administration of the GFAP-TH plasmid DNA targeted with either the TfRMAb or the mouse IgG2a isotype control antibody, was 4 ± 3 rotations per min (RPM) and 22 ± 3 RPM, respectively, following the administration of apomorphine (Zhang et al., 2004a). The lack of any therapeutic effect following the administration of THLs, encapsulating the GFAP-TH plasmid DNA, but targeted only with the mouse IgG2a isotype control antibody, indicates the delivery of THLs to neurons in the brain is strictly a function of the receptor specificity of the targeting MAb conjugated to the THL. When the TH gene was placed under the influence of the GFAP promoter, off-target effects were eliminated, and no hepatic expression of TH was observed following the IV administration of TfRMAb-targeted THLs (Zhang et al., 2004a).

The GFAP promoter enabled TH gene expression in neurons in the PD model (Zhang et al., 2004a), and early work showed that the 5′-FS of the GFAP gene confers brain specificity, but not astrocyte specificity of gene expression. Astrocyte specificity of gene expression is produced by coordinate interactions between regulatory elements in both the 5′-FS and the 3′-FS of the GFAP gene (Kaneko and Sueoka, 1993; Galou et al., 1994). The GFAP 5′-FS alone enables neuronal expression in transgenic mouse models (Zhuo et al., 2001).

Gene therapy aimed at the replacement of the deficient TH enzyme activity in the striatum of PD does not block or slow the neurodegeneration of PD. Neurotrophins, such as GDNF, can slow or block the nigrostriatal neurodegeneration of PD. Therefore, the THL technology was applied to experimental PD following the engineering of a human prepro GDNF expression plasmid under the influence of the striatal specific promoter taken from the 8 kb of the 5′FS of the rat TH promoter (THpro; Zhang and Pardridge, 2009). The PD lesion was introduced by the unilateral injection of 8 μg of 6-hydroxydopamine into the right MFB, and weekly THL gene therapy was started 3 days after toxin injection and continued for 3 weeks. The rotational motor activity induced by either apomorphine, which causes rotations contralateral to the toxin injection side, or amphetamine, which causes rotations ipsilateral to the toxin injection site, was monitored for 6 weeks after toxin injection. The rotational activity after the injection of apomorphine or amphetamine is shown in the left and right panels, respectively in Figure 8E. In the toxin lesioned rats, treated only with saline, the motor activity continued to deteriorate over the 6 weeks following the toxin injection. However, in the toxin lesioned rats treated with the THpro-preproGDNF plasmid DNA encapsulated in TfRMAb-targeted THLs, there was a near abrogation of aberrant motor activity (Figure 8E). The improvement in motor activity with 3 weeks of THL gene therapy was correlated with a 77% normalization of striatal TH enzyme activity (Zhang and Pardridge, 2009).

### THL Gene Therapy of Brain Cancer and RNA Interference

The RNAi enables selective suppression of target mRNAs and is an attractive therapy of brain cancers that are dependent on certain oncogenes. Glioblastoma multiforme (GBM) is the most aggressive type of brain cancer and is accelerated by over-expression of the epidermal growth factor receptor (EGFR; Kuan et al., 2001). There are two types of RNAi therapeutics that might be developed to suppress the EGFR mRNA in GBM: (i) RNA-based RNAi with short interfering RNA (siRNA); and (ii) and plasmid DNA-based RNAi that express short hairpin RNA (shRNA). There are no FDA approved RNAi drugs for brain cancer, or for any other brain disease, because the fundamental problem of BBB delivery of RNAi drugs has not been solved. In DNA-based RNAi, a plasmid DNA is delivered to the brain, followed by the expression of a sequence-specific shRNA within the cancer cell that targets the EGFR mRNA. The THL delivery of DNA-based RNAi therapy was evaluated in a model of brain cancer that over-expressed the EGFR (Zhang et al., 2004b). The sequence within the human EGFR mRNA that was optimal for the induction of RNAi was determined following the engineering of a series of 6 shRNA encoding expression plasmids, which target different sequences of the human EGFR mRNA (accession number X00588). The plasmid sequence encoding the shRNA was downstream of the U6 RNA polymerase and upstream of an oligo-deoxythymidine termination sequence. An examination of the effect of lipofection with these six expression plasmids on [^3^H]-thymidine incorporation in U87 human glioma cells showed the maximal suppression of cell growth was obtained with a plasmid DNA that expressed an shRNA that targeted nucleotides 2,529–2,557 of the human EGFR mRNA, and this expression plasmid was designated clone 967 (Zhang et al., 2004b). The reduction in EGFR-mediated function in cultured U87 tumor cells by treatment with the clone 967 plasmid DNA encapsulated within THLs targeted with the HIRMAb was demonstrated by measurement of intracellular calcium flux in these tumor cells treated with the EGF peptide. Following THL RNAi treatment for 24 h, the U87 cells exhibited a >90% suppression of change in intracellular calcium flux in response to the EGF peptide (Zhang et al., 2004b).

**Figure 9:**
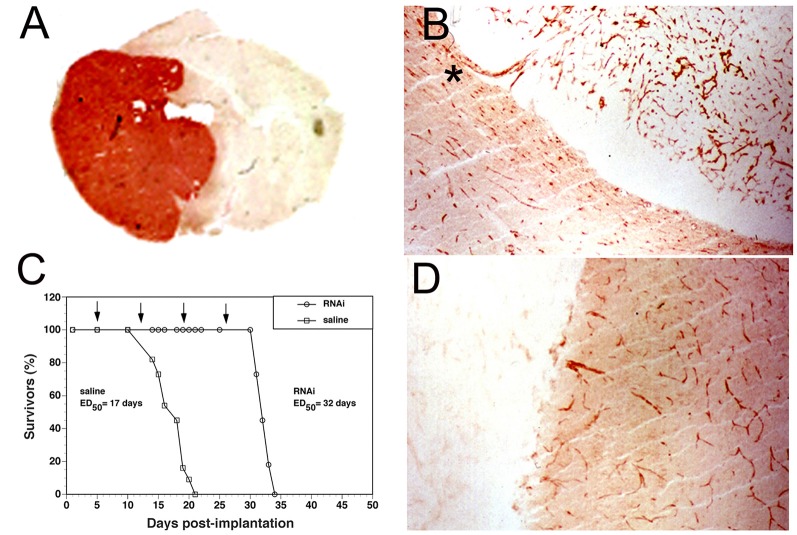
**(A)** Coronal section of the brain of severe combined immunodeficient (SCID) mouse at expiration following the implantation of 500,000 human U87 glioma cells in the caudate-putamen nucleus (CPN), and immunostained with the 528 monoclonal antibody (MAb) against the human epidermal growth factor receptor (EGFR). Panel **(A)** reprinted by permission from Zhang et al. (2002). **(B)** Section of SCID mouse brain at expiration after the implantation of 500,000 human U87 glioma cells in the CPN, and immunostained with the 8D3 MAb against the mouse transferrin receptor (TfR). The brain tumor-bearing mice were treated with weekly injections of saline starting 5 days after tumor implantation. Capillaries originating from normal mouse brains are seen vascularizing the human U87 tumor (*). **(C)** Survival of SCID mice following the implantation of 500,000 human U87 glioma cells in the CPN at day 0. Starting at 5 days after tumor implantation, the mice were treated with weekly IV injections of either saline or THLs doubly targeted with the 8D3 MAb against the mouse TfR, and the 83–14 MAb against the HIR. The THLs encapsulated a plasmid DNA that encoded a 29 nucleotide (nt) short interfering RNA (shRNA) that targeted nucleotides 2,529–2,557 of the human EGFR mRNA. The plasmid expression of the shRNA leads to RNA interference (RNAi) of the EGFR transcript in the brain tumor. The weekly injection dose of plasmid DNA was 5 μg/mouse. **(D)** Section of SCID mouse brain at expiration after the implantation of 500,000 human U87 glioma cells in the CPN, and immunostained with the 8D3 MAb against the mouse transferrin receptor (TfR). Starting at 5 days after tumor implantation, the mice were treated with weekly injections of the THLs doubly targeted with the TfRMAb and HIRMAb and encapsulating the plasmid DNA encoding the human EGFR mRNA-specific shRNA. There is an 80% reduction in capillary density in the tumor of the RNAi treated mice, as compared to the saline-treated mice (panel **B**). Panels **(B–D)** reprinted by permission from Zhang et al. (2004b).

The *in vivo* therapeutic effects of THL delivery of shRNA encoding plasmid DNA (clone 967) were examined in a mouse intracranial human brain cancer model. Human U87 glioma cells (500,000), which overexpress the EGFR, were implanted, under stereotactic guidance, in the caudate-putamen nucleus (CPN) on one side of the brain of 20 g female SCID mice (Zhang et al., 2004b). By 5 days after implantation of this number of U87 cells, the entire volume of the CPN is filled with cancer (Lal et al., 2000). At death, the entire hemisphere of the mouse brain is nearly filled with the cancer, as shown in Figure 9A, which is an immunocytochemical study using the 528 MAb against the human EGFR. The clone 967 expression plasmid was encapsulated in THLs that were targeted with the 8D3 MAb against the mouse TfR, to enable RMT across the capillary endothelium perfusing the tumor. Microvessels from normal mouse brain vascularize the human U87 tumor (see * in Figure 9B). Both the microvessels of the normal mouse brain and the microvessels perfusing the U87 human glioma expressed the mouse TfR (Figure 9B). However, while the 8D3 TfRMAb reacts with the mouse TfR within mouse neuropil, this mouse TfR-specific MAb does not recognize the human TfR on human U87 cells (Figure 9B). Therefore, so as to enable the THL to traverse the human tumor cell membrane, the THL was also targeted with the 83–14 murine MAb against the HIR. The doubly targeted THL is able to cross the mouse BBB, *via* the TfRMAb, and is also able to cross the human tumor cell membrane, *via* the HIRMAb (Zhang et al., 2004b). Weekly IV treatment of the mice with the TfRMAb/HIRMAb-targeted THL, encapsulating the clone 967 plasmids DNA, was initiated at 5 days following tumor implantation in the brain. A control group of tumor-bearing mice was treated with saline. The saline-treated mice were 50% dead at 17 days, and 100% dead at 21 days after tumor cell implantation. The survival time of the THL treated mice was increased by nearly 90%, and these mice were 50% dead at 32 days, and 100% dead at 34 days (Figure 9C). The death of the RNAi-treated mice was associated with a therapeutically induced suppression of the vascular density within cancer. The EGFR has a pro-angiogenic effect in brain cancer (Abe et al., 2003). Consequently, RNAi-suppression of the tumor EGFR has an anti-angiogenic effect within the tumor that is not observed in the normal brain (Zhang et al., 2004b). The capillary density in normal brain, 35 ± 1 capillary per 0.1 mm^2^ of the brain, was unchanged by the THL RNAi treatment. However, the capillary density within the brain tumor, which was 15 ± 2 capillary per 0.1 mm^2^ in the saline-treated mice, was reduced 80% in the RNAi treated mice (Zhang et al., 2004b). The marked reduction in capillary density in the tumor of the RNAi-treated mice is shown by the immunocytochemical study in Figure 9D, which detects the capillaries immunopositive with the 8D3 MAb against the mouse TfR. A normal density of capillaries is visible in the non-tumor brain, whereas the capillary density in the brain cancer is greatly reduced as compared to the tumor capillary density in saline-treated mice (Figure 9B). Confocal microscopy of the terminal brain tumors demonstrated a marked reduction in the EGFR protein in the RNAi-treated mice as compared to the saline-treated mice (Zhang et al., 2004b).

The tumor escape from THL-mediated RNAi therapy ultimately led to the death of the tumor-bearing mice (Figure 9C). Similar to classical chemotherapy of cancer with a cocktail of small molecule drugs, the treatment of brain cancer with RNAi-based drugs might be optimized by the administration of different plasmid DNAs that target multiple oncogenic proteins within cancer. The RNAi brain tumor model shown in Figure 9 illustrates it is possible to knock down pathologic genes in the brain with plasmid DNA-based RNAi therapy coupled with the THL targeting technology.

### Translation of the THL Technology to Humans

The future translation of the THL technology to the treatment of brain disease in humans must address both safety of chronic THL administration and a scalable THL manufacturing process. With regard to safety, the HIRMAb that would be used to target THL delivery across the human BBB has already been used in human clinical trials. Subjects with MPSI were treated with doses up to 6 mg/kg of an HIRMAb-IDUA fusion protein for over 1 year with a favorable safety profile (Giugliani et al., 2018). The delivery of plasmid DNA to the Rhesus monkey brain is enabled with an IV dose of the HIRMAb that is much lower, 0.3 mg/kg (Zhang et al., 2003c), as compared to the HIRMAb doses administered in the 1 year clinical trial of MPSI (Giugliani et al., 2018). THLs have been administered IV to rats chronically on a weekly basis without any evidence of toxicity (Zhang Y. F. et al., 2003). Off-target effects of gene expression can be minimized with the use of tissue-specific gene promoters that limit transgene expression to a target region (Zhang et al., 2003b). THL-mediated gene expression is reversible, which is considered an advantage in first-generation gene therapeutics. Once the stable integration of the human genome, without insertional mutagenesis, is possible, the plasmid DNA can be engineered to enable integration of the transgene within the human genome.

The scalable manufacturing of THLs presents the greatest challenge. The mass production of the recombinant HIRMAb or the plasmid DNA is now routine. However, with respect to liposome manufacturing, the thin film/extruder process used to manufacture THLs for preclinical investigations is not scalable. The production of DNA encapsulated pegylated liposomes with the ethanol dilution process is scalable (Jeffs et al., 2005), and could meet market demand at least for orphan diseases of the brain. A scalable THL manufacturing process would also need to develop an effective cryoprotectant to enable freeze-drying of the THLs to sustain a commercially acceptable shelf life of the pharmaceutical.

## Conclusions and BBB Avoidance Strategies

The development of new drugs for brain diseases, much less the major diseases of the brain in aging, AD, PD, and stroke, has proven to be most difficult, particularly for biologic drugs. In 2019, there is not a single recombinant protein that is FDA approved for brain disease, wherein that drug must cross the BBB. The main factor limiting CNS drug development is the BBB, as 98% of all small molecules do not cross the BBB, and ~100% of large molecule drugs do not cross the BBB (Pardridge, 2005). Multiple clinical trials of CNS disease have been attempted with recombinant proteins over the last 25 years, and all such clinical trials have failed (Pardridge, 2015a). The singular feature of all these trials is that, in no case, was the biologic re-engineered to enable BBB transport prior to entry into the human clinical trial. This is a natural result of CNS drug development taking place in the absence of a parallel effort in BBB drug delivery technology.

The fact that multiple biologics have entered CNS clinical trials over the last 25 years, without any BBB drug delivery technology, seems paradoxical. These clinical trials were enabled because CNS drug developers practiced a variety of BBB avoidance strategies, wherein the brain drug delivery strategy emanated not from a foundation in BBB drug delivery technology, but rather from a basket of BBB avoidance strategies that: (i) asserted the drug crossed the BBB, (ii) employed BBB disruption strategies, (iii) bypassed the BBB by drug injection into the brain or CSF; or (iv) employed ineffective BBB delivery vehicles, such as stem cells or AAV. Such BBB avoidance strategies are generally not effective, and with rare exceptions, the clinical trials lead predictably to failure and no FDA approval. The top 10 BBB avoidance strategies are highlighted below.

1. **Use of drug entry into CSF as an index of drug transfer across the BBB**. Therapeutic antibody-drug developers for brain claim no BBB delivery technology is needed, owing to a low, but significant, antibody delivery into the brain from the blood. The IgG concentration in the brain is said to be 0.1%–0.2% of the blood IgG concentration (Atwal et al., 2011; Bohrmann et al., 2012). However, what is being cited in this context is the concentration of antibodies in CSF, not the brain. Drug transport from blood into CSF is a function of delivery across the choroid plexus, which forms the blood-CSF barrier, whereas drug transport from blood into brain parenchyma is a function of delivery across the brain capillary endothelium, which forms the BBB (Pardridge, 2016). The choroid plexus epithelial barrier and the brain endothelial barrier are anatomically and functionally distinct. The blood-CSF barrier is much leakier than is the BBB. All proteins in blood enter into CSF across the leaky choroid plexus, at a rate inversely related to the molecular weight of the drug (Reiber, 2003). The concentration of IgG in CSF is 0.1%–0.2% of the corresponding plasma level (Reiber, 2003), whereas the concentration in brain parenchyma of a therapeutic antibody is <0.01% of the plasma concentration (Yadav et al., 2017). Drug penetration into CSF, across the choroid plexus, is not a surrogate marker of drug penetration into the brain parenchyma, across the BBB, and antibody distribution into CSF should not be used as a rationale for CNS antibody drug development without BBB drug delivery technology.
2. **Failure to account for the cerebral blood volume**. Aducanumab, the therapeutic anti-Abeta amyloid antibody, was said to cross the BBB because the brain concentration was parallel to the plasma concentration in preclinical research in mice (Sevigny et al., 2016). However, the brain/plasma ratio of this antibody was 1 μl/g, which is >10-fold lower than the brain plasma volume, 10–15 μl/g (Pardridge, 2019). Antibody residing in residual blood volume of brain has not crossed the BBB. For assessment of biologic uptake by the brain, it is important to correct brain drug uptake estimates for drugs trapped in the blood volume of the brain. Failure to correct for the brain-blood volume may lead CNS antibody-drug developers to conclude that no BBB delivery technology is required.
3. **Drug-induced BBB disruption**. Aducanumab reduces brain amyloid plaque in AD in a dose-dependent mechanism, which indicates this antibody crossed the BBB in patients with AD (Sevigny et al., 2016). However, aducanumab also caused a dose-dependent disruption of the BBB, as reflected in the vasogenic edema measured in these patients by MRI (Sevigny et al., 2016). The BBB disruption in AD caused by high doses of an anti-amyloid antibody parallels the cerebral micro-hemorrhage observed in transgenic AD mice administered high doses of an anti-amyloid antibody (Wilcock et al., 2004). The AAA that do not cause BBB disruption or brain edema in AD also do not lower brain amyloid plaque (Cummings et al., 2018; Salloway et al., 2018).
4. **Drug injection into the CSF**. Over 100 years ago, when a barrier between brain and blood was just discovered, it was believed that nutrients in blood passed first into CSF and then into the brain (Pardridge, 2016). These ideas were soon shown to be false and that drug entered the brain directly from blood without any intermediate passage through the CSF. Nevertheless, these ideas gave rise to the concept that drug injected into CSF easily distributed to the brain. However, a drug injected into CSF does not undergo significant penetration into the brain parenchyma (Figure 2), because drug diffusion into the brain from the CSF is slow compared to the rapid bulk flow of CSF out of the brain to the peripheral blood. A drug injection into the spinal fluid is equivalent to a slow IV injection (Fishman and Christy, 1965). Intrathecal drug administration is useful when the disease target is on the surface of the brain or spinal cord, such as meningeal carcinoma (Larson et al., 2015), or drug delivery to the motor neurons near the surface of the lumbar spinal cord. Nusinersen (Spinraza^®^) is a phosphorothioate antisense oligonucleotide that is FDA approved for the treatment of SMA (Neil and Bisaccia, 2019). The drug is injected directly into the lumbar CSF, where the affected motor neurons within the anterior horn of the spinal cord sit just 1–2 mm from the CSF bathing the surface of the spinal cord (Bican et al., 2013).
5. **Intra-cerebral drug injection into brain tissue**. The drug may be injected into the brain through a Burr hole drilled in the skull. However, drug distribution is confined largely to the tip of the injection needle (Mastakov et al., 2001), owing to the limitation of diffusion within the brain. Convection enhanced diffusion (CED) is an attempt to enhance drug penetration through brain parenchyma by a pump driven convection process (Lang et al., 2006). However, once the fluid exits the infusion catheter, the resistance of the brain tissue limits convection, and drug penetration into the brain decreases logarithmically from the catheter tip (Salvatore et al., 2006), which is indicative of diffusion, not convection.
6. **Nasal delivery to the brain**. The same rules that govern drug transport across the BBB also determine drug delivery across the nasal barriers: (a) lipid-soluble small molecules cross by free diffusion; and (b) water-soluble small molecules or biologics do not cross in the absence of membrane injury. Lipid soluble small molecules do enter olfactory CSF following nasal delivery, because the small molecule is able to first diffuse across the nasal epithelial barrier and then diffuse across the olfactory arachnoid membrane. In experimental settings where large molecule drugs enter olfactory CSF and then brain, this is invariably associated with the nasal instillation of large volumes of fluid, which cause local injury to nasal membranes (Merkus et al., 2003). Injury-based drug delivery strategies cannot be translated to humans.
7. **Stem cell delivery to the brain**. Stem cells are often assumed to cross the BBB, which forms the basis of treatment of certain orphan diseases of the brain with IV HSC transplant. However, microscopic examination of brain following IV administration of stem cells shows that stem cells do not cross the BBB and do not enter brain parenchyma (Soper et al., 2004). Stem cells do invade the meninges of the brain (Soper et al., 2004), which are outside the BBB.
8. **AAV viral delivery to the brain**. The IV administration of certain AAV serotypes, e.g., AAV9, results in transvascular viral delivery to the brain (Foust et al., 2009). However, only up to ~20% of brain cells are transduced, and only at high injection doses of 10^14^ vg/kg of self-complementary forms of AAV (Foust et al., 2009; Hudry et al., 2018). This high injection dose of 10^14^ vg/kg has been administered in human clinical trials (Mendell et al., 2017). AAV injection doses at the 10^14^ vg/kg level are associated with a high incidence of delayed liver cancer in mice (Chandler et al., 2015).
9. **Transitory BBB disruption**. Transient drug or even gene delivery to the brain is possible with ultrasonic irradiation of the brain following the IV administration of micro-bubbles (Chang E. L. et al., 2017). The combination of the ultrasonic irradiation of a targeted region of the brain and the presence of the microbubbles in the blood causes transient BBB disruption. Transitory BBB disruption may be useful for the treatment of regional diseases such as brain cancer. However, chronic BBB disruption is injurious to the brain and induces neurodegeneration (Salahuddin et al., 1988).
10. **Small molecule drug development**. A new neurotrophin may be discovered for AD, and it is recognized that this neurotrophin does not cross the BBB. What is proposed is the discovery of a small molecule peptidomimetic based on rational drug design (Kazim and Iqbal, 2016). However, rational drug design invariably leads to the discovery of drugs that are either water-soluble, e.g., form ≥8 hydrogen bonds with water, and/or have a molecular weight (MW) >400 Da, which exceeds the MW threshold for small molecule transport across the BBB (Pardridge, 2005). Most drugs lack the dual characteristics of lipid solubility and MW <400 Da, which is why only 1% of all small-molecule drugs are active in the CNS, excluding affective disorders (Lipinski, 2000). Small molecule CNS drug developers have to deal with the BBB delivery problem nearly to the same extent as large molecule CNS drug developers.

Avoidance of the development of BBB drug delivery technology is not the way to solve the brain drug and gene delivery problem. The future development of new biologic treatments of AD, and other brain diseases, that are FDA approvable, will require a concerted effort in the innovation of new BBB drug delivery technology platforms. Such technology invariably is focused on the use of endogenous CMT systems, in the case of small molecule transport, or the endogenous RMT systems, in the case of biologics (Figure 3). Without an effort in BBB drug delivery that is equal to the ongoing effort in CNS drug discovery, then the current abysmal rate of FDA approval of drugs for the brain, including AD, is not expected to change well into the future.

## Author Contributions

WP wrote the manuscript.

## Conflict of Interest

The author is consultant to ArmaGen, Inc., and The Lipogene Company, Inc., and inventor of issued patents on brain drug delivery of biologic and gene medicines.
